# Multiple freshwater invasions of the tapertail anchovy (Clupeiformes: Engraulidae) of the Yangtze River

**DOI:** 10.1002/ece3.5708

**Published:** 2019-10-01

**Authors:** Fangyuan Cheng, Qian Wang, Pierpaolo Maisano Delser, Chenhong Li

**Affiliations:** ^1^ Shanghai Universities Key Laboratory of Marine Animal Taxonomy and Evolution Shanghai China; ^2^ Shanghai Collaborative Innovation for Aquatic Animal Genetics and Breeding Shanghai China; ^3^ Key Laboratory of Exploration and Utilization of Aquatic Genetic Resources Ministry of Education Shanghai Ocean University Shanghai China; ^4^ Department of Zoology University of Cambridge Cambridge UK; ^5^ Smurfit Institute of Genetics Trinity College University of Dublin Dublin Ireland

**Keywords:** *Coilia nasus* species complex, ecotypes, freshwater adaptation, paleogeography, population genomics, systematics

## Abstract

Freshwater fish evolved from anadromous ancestors can be found in almost all continents. The roles of paleogeographic events and nature selection in speciation process often are under focus of research. We studied genetic diversity of anadromous and resident tapertail anchovies (*Coilia nasus* species complex) in the Yangtze River Basin using 4,434 nuclear loci, and tested the history of freshwater invasion of *C. nasus*. We found that both *C. brachygnathus* and *C. nasus* were valid species, but the resident *C. nasus taihuensis* and the anadromous *C. nasus* were not different genetically based on Bayes factor species delimitation (BFD*). Maximum likelihood tree, Network, PCA and STRUCTURE analyses all corroborated the results of BFD*. Two independent freshwater invasion events of *C. nasus* were supported, with the first event occurring around 4.07 Ma and the second happened around 3.2 Ka. The time of the two freshwater invasions is consistent with different paleogeographic events. Estimation showed that gene flow was higher within ecotypes than between different ecotypes. F‐DIST analyses identified 120 disruptive outliers by comparing *C. brachygnathus* to anadromous *C. nasus*, and 21 disruptive outliers by comparing resident *C. nasus* to anadromous *C. nasus*. Nine outliers were found to be common between the two comparisons, indicating that independent freshwater invasion of *C. nasus* might involve similar molecular pathways. The results of this study suggest that adaptation to landlocked freshwater environment of migratory fish can evolve multiple times independently, and morphology of landlocked ecotypes may cause confusion in their taxonomy.

## Graphical abstract

We studied genetic diversity of anadromous and resident tapertail anchovies (*Coilia nasus* species complex) in the Yangtze River Basin using 4,434 nuclear loci, and found that both *C. brachygnathus* and *C. nasus* were valid species, but the resident *C. nasus* taihuensis and the anadromous *C. nasus* were not different genetically. Two independent freshwater invasion events of *C. nasus* were supported, with the first event occurring around 4.07 Ma and the second happening around 3.2 Ka.

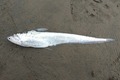

## 1 INTRODUCTION

Freshwater invasion of marine fishes can be found in many water systems across almost all continents. The species that successfully invaded freshwater are phylogenetically sporadic, clustered in clades such as sticklebacks, marine catfishes, puffer fishes, gobies, herrings, and anchovies (Bell & Foster, 1993; Betancur, Orti, Stein, Marceniuk, & Alexander Pyron, 2012; Bloom & Lovejoy, 2012; Cooke, Chao, & Beheregaray, 2012; Michel et al., 2008; Palkovacs, Dion, Post, & Caccone, 2008; Wilson, Teugels, & Meyer, 2008). Genetic and phenotypic traits of invaded species often were changed due to adaptation to new environments (Cooke et al., 2012; Palkovacs et al., 2008). Transitions from marine to freshwater habitats initiated the radiation and speciation of many taxa (Lee & Bell, 1999). Moreover, paleogeographic events might also have played a major role in shaping genetic structure of species invading freshwater (Bloom & Lovejoy, 2012; Wilson et al., 2008).

**Figure 1:**
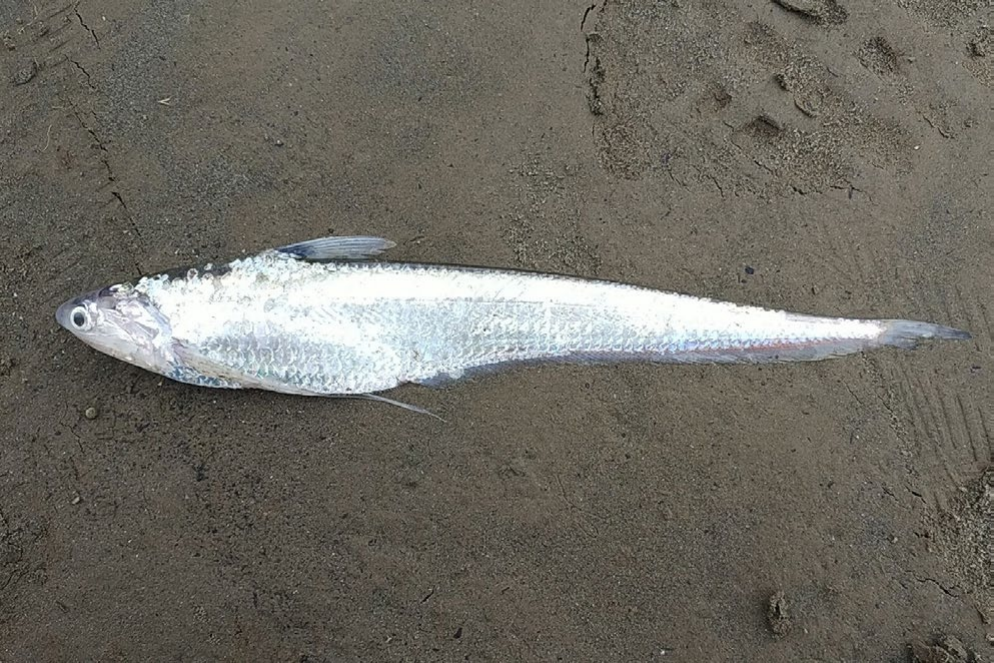
A migratory *Coilia nasus* specimen collected from the main channel of the Yangtze River at Chongming, Shanghai

The tapertail anchovies are distributed along coastal waters of the Indo‐West Pacific, often frequenting estuaries and tolerating lowered salinities (Whitehead, Nelson, & Wongratana, 1988). In the Yangtze River Basin, there are two groups of tapertail anchovies, *Coilia mystus* and *Coilia nasus* species complex. *Coilia mystus* enters estuary of the Yangtze River for spawning but never further upstream into freshwater (Ni & Wu, 2006). *Coilia nasus* species complex has several ecotypes, subspecies, or species according to different studies (Liu, 1995; Tang, Hu, & Yang, 2007; Yuan, Lin, Qin, & Liu, 1976; Yuan, Qin, Liu, & Lin, 1980), including the anadromous *C. nasus* (Figure 1), freshwater resident *C. nasus taihuensis*, and *C. brachygnathus*.

*Coilia nasus* (Temminck & Schlegel, 1846) was originally described from a specimen collected in Japan. Jordan and Seale (1905) subsequently described a fish collected from Shanghai as *Coilia ectenes*, which should be a synonym of *C. nasus* (Ni & Wu, 2006). Kreyenberg and Pappenheim (1908) described a new species, *C. brachygnathus*, from a sample collected in Lake Dongting of the Yangtze River Basin. A short maxilla, not reaching to edge of gill cover, was used to diagnose *C. brachygnathus* (Kreyenberg & Pappenheim, 1908; Whitehead et al., 1988; Yuan et al., 1980).

Fishermen of Lake Chao and Lake Tai have long recognized that resident *C. nasus* caught from those lakes was different from the anadromous fish of main channels of the Yangtze River (Yuan et al., 1976). Morphological difference between these two ecotypes, such as average number of vertebrae, average number of anal fin rays, ratio of snout length to eye diameter, and length of liver, was used to describe the resident ecotypes of Lake Chao and Lake Tai as a new subspecies *C. nasus taihuensis* (Yuan et al., 1976).

**Figure 2:**
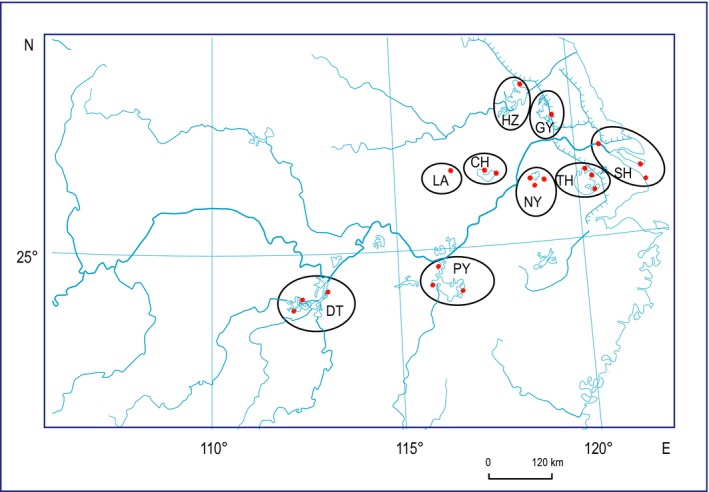
Geographic range of each population (circles) and collection sites (dots) at: Lake Dongting (DT), Lake Poyang (PY), Lake Nanyi (NY), Lake Tai (TH), Luan (LA), Lake Chao (CH), Lake Hongze (HZ), Lake Gaoyou (GY), main channels, and estuary area of the Yangtze River at Chongming, Luchao, and Jingjiang (SH)

*Coilia nasus* lives in coastal and estuarial regions of the Northwest Pacific Ocean and migrates upstream into freshwater for breeding. In the Yangtze River Basin, it moves upstream into the river and associated freshwater lakes for spawning, as far as to Lake Dongting. *Coilia nasus taihuensis* is a freshwater resident, living its whole life in lakes associated with lower reaches of the Yangtze River, such as Lake Tai and Lake Chao (Yuan et al., 1980). *Coilia brachygnathus* is also a resident fish, but it is distributed in lakes connected to further upstream of the Yangtze River, such as Lake Poyang and Lake Dongting (Figure 2).

Early taxonomic studies provided diagnostic characters for different ecotypes and three species/subspecies were established (Yuan et al., 1976, 1980), but some later morphometric and molecular studies suggested that all three species/subspecies belong to a single species, *C. nasus* (Cheng & Han, 2004; Liu, 1995; Tang et al., 2007). This controversy probably is rooted in plasticity of the morphological characters and morphometric traits used for taxonomy of the *C. nasus* species complex. For example, the diagnostic character of *C. brachygnathus*, a short maxilla, was also found in *C. nasus taihuensis* of Lake Tai and *C. nasus* collected from main channels of the Yangtze River (Ni & Wu, 2006; Tang et al., 2007). Those traits might be adaptations to freshwater conditions.

Recent molecular studies revealed low level of genetic divergence between *C. nasus*, *C. nasus taihuensis*, and *C. brachygnathus* (Cheng, Zhang, Ma, & Guan, 2011; Zhou, Yang, Tang, & Liu, 2010). *Coilia nasus*, *C. nasus taihuensis*, and *C. brachygnathus* did not form reciprocal monophyletic clades (Tang et al., 2007) and the genetic p‐distance among the three taxa was between 0.253% and 0.557% (Cheng et al., 2011). Nevertheless, most of those molecular studies were based on a single mitochondrial locus. Moreover, no hypotheses were tested explicitly about the history of freshwater invasions of the *C. nasus* species complex in the Yangtze River Basin. The paleo‐Yangtze drainage was fragmented by the north‐south trending Wushan mountain range in the middle and the southeast coastal mountain range in the east part of the present Yangtze drainage basin (Fan & Li, 2008). Only rudimental river system of the modern Yangtze River originating from the southeast coastal mountain drained into the East China Sea (Wang, 1985). Chemical dating by electron microprobe showed that the modern Yangtze draining the Tibetan plateau to East China Sea should have formed before 2.58 Ma (Fan & Li, 2008; Fan, Li, & Yokoyama, 2005). During the subsequent glacial age, sea level was lower and the main stream of the Yangtze River had eroded downward, and lakes associated with the middle‐lower Yangtze River were dried up. Postglacially, water level of the Yangtze River had risen up and development of the modern lakes started (Yang, Li, & Zhang, 2000). Those paleogeographic changes should have altered evolutionary history of fishes in the Yangtze River and affected freshwater invasion of *C. nasus*.

In this study, we collected genome‐scale nuclear sequence data (4,434 loci) applying a cross‐species target gene capture approach (Li, Hofreiter, Straube, Corrigan, & Naylor, 2013) and examined samples from all major lakes of the Yangtze River Basin as well as its main channels. We aimed to test: (a) whether freshwater invasion happened once or multiple times in the *C. nasus* species complex of the Yangtze River Basin; (b) what are the genetic changes that resulted from freshwater invasion, and whether those changes are shared or not between independently invaded freshwater populations; and (c) should the ecomorphs be recognized as one species or three if evaluated using genome‐scale data.

## 2 MATERIALS AND METHODS

### 2.1 Sample collection and DNA extraction

A total of 138 samples of *C. nasus* species complex were collected from 11 lakes, river branches, or channels of the Yangtze River Basin (Figure 2 and Table 1), including Lake Dongting (DT, *n* = 18, from three locations), Lake Poyang (PY, *n* = 21, from three locations), Lake Nanyi (NY, *n* = 19, from three locations), Luan (LA, *n* = 9), Lake Chao (CH, *n* = 19, from two locations), Lake Hongze (HZ, *n* = 10), Lake Gaoyou (GY, *n* = 8), Lake Tai (TH, *n* = 21, from three locations), main channel of the Yangtze River at Jingjiang (*n* = 3), the Yangtze River around Chongming Island (*n* = 5), and coast of Shanghai at Port Luchao (*n* = 5). The 13 samples collected from Chongming, Luchao, and Jingjiang were considered as the anadromous population (SH, *n* = 13), whereas the other samples were treated as the resident populations. Five samples of *C. mystus* collected from the Yangtze River were used as outgroups, since it was found closely related to *Coilia nasus* species complex (Lavoué et al., 2017). Most samples of the resident populations were collected after October, when the migratory fish had already left freshwater habitat. The resident ecotype also was confirmed by checking strontium to calcium ratio (Sr/Ca) of sagittal otoliths of the sampled fish. The ratio of Sr/Ca can be used to reconstruct habitat use of individual fish (Yang, Arai, Liu, Miyazaki, & Tsukamoto, 2006), so it was applied to verify resident ecotype of our sampled fish. Multiple sites within each lake were sampled to provide a better representation of the genetic diversity of the fish population for each lake (Table 1). Fin clips or muscle tissues were taken from the fish and preserved in 95% ethanol under 4°C. Total genomic DNA was extracted using a tissue DNA kit (Omega Bio‐tek). The extracted DNA was quantified using NanoDrop 3300 Fluorospectrometer (Thermo Fisher Scientific).

**Table 1:** Sample collection, 138 samples of the *Coilia nasus* complex and five samples of *Coilia mystus* as outgroup

Water body	Sample ID	Locality	No. of samples	Total	Date of collection
Lake Dongting (DT)	CL452	Yue‐Yang	8	18	2013/4/22
	CL1257	Yuan‐Jiang	4		2016/9/8
	CL1237	Yuan‐Jiang	6		2016/11/4
Lake Poyang (PY)	CL1255	De‐An	9	21	2016/9/4
	CL1238	De‐An	5		2016/11/8
	CL1239	Po‐Yang	7		2016/11/8
Lake Nanyi (NY)	CL375	Xuan‐Cheng	5	19	2013/4/7
	CL1241	Nan‐Yi‐Hu	7		2016/11/28
	CL1242	Lang‐Xi	7		2016/11/28
Luan (LA)	CL410	Lu‐An	9	9	2013/4/10
Lake Chao (CH)	CL1256	Ju‐Chao	10	19	2016/9/6
	CL1240	Chao‐Hu	9		2016/11/6
Lake Tai (TH)	CL507	Guang‐Fu	8	21	2013/11/22
	CL1235	Wu‐Xi	6		2016/11/3
	CL1236	Su‐Zhou	7		2016/11/3
Lake Hongze (HZ)	CL1164	Si‐Hong	10	10	2016/7/22
Lake Gaoyou (GY)	CL506	Gao‐You	8	8	2013/11/12
Main channels and estuary of the Yangtze River (SH)	CL540	Chong‐Ming	5	13	2013/4/1
	CL63	Lu‐Chao‐Gang	1		2012
	CL519	Lu‐Chao‐Gang	4		2013/11/23
	CL64	Jing‐Jiang	1		2013
	CL66	Jing‐Jiang	1		
	CL68	Jing‐Jiang	1		
Outgroup (*Coilia mystus*)	CL81‐84, CL447_7	Shanghai	5	5	2011

### 2.2 Sr/Ca ratio of sagittal otoliths

Thirty‐two fish from seven lakes (4–5 for each lake) and one fish from an anadromous population were analyzed for relative composition of trace element (Sr/Ca) of their otoliths. The otoliths were placed into a rectangular plastic mold and embedded in epoxy resin (Epofix; Struers). The otoliths were subsequently grinded with sand paper of 240 grits, 600 grits, 1,200 grits, and 2,000 grits in turn on Struers grinder (Discoplan‐TS, Struers), until the core of sagittal plane was exposed. The ratio of Sr/Ca was determined by coupled plasma mass spectrometer (ICPMS, Agilent 7700x, Agilent Technologies), in center, margin, and middle position of the sagittal otoliths.

### 2.3 DNA library preparation, gene capture and sequencing

The DNA samples were sheared using a Covaris M220 sonicator (Gene Company Limited, Covaris) to about 500 bp according to the manufacturer's instructions. The size of sheared DNA was checked by agarose gel electrophoreses. DNA libraries were constructed following Li et al. (2013). Inline indices were added in the ligation step of library preparation to label each sample to reduce potential risk of cross contamination between samples during subsequent gene capture steps. The inline indices are 6 bp nucleotides attached to 3′ prime end of regular Illumina IS1 and IS3 oligos. Each inline index is separated by two different nucleotides from the others. The inline‐index‐coded DNA libraries were pooled together equimolarly for subsequent gene capture (~18 samples per group).

A suite of 4,434 target loci were developed for ray‐finned fishes by Jiang et al. (2019). Sequences of the 4,434 loci of two clupeiform species, *Denticeps clupeoides* and *Ilisha elongata*, collected in our previous experiments were used in designing RNA baits. The sequences of *D. clupeoides* were preferred for bait design, because its DNA has lower GC content (~50%) than that of *I. elongata* (70%–80%). The sequence of *I. elongata* was used for bait design only if it was not found in *D. clupeoides* for some loci. The target sequences used for bait design can be retrieved in Dyrad (https://doi.org/10.5061/dyrad.2j5b4). MYbaits RNA probes targeting the 4,434 loci were synthesized at Arbor Biosciences (cat#: ClupiformsV2 MYbaits‐1).

A cross‐species gene capture approach was followed (Li et al., 2013). The samples were captured twice as recommended. The enriched libraries were amplified with IS4 and indexing primers with 8 bp DNA barcodes following Meyer and Kircher (2010). The final products were pooled equimolarly and sequenced in one lane of an Illumina HiSeq 2500 flow cell (Anoroad Genome).

### 2.4 Read assembly and SNP calling

Raw reads were parsed to each sample according the 8 bp barcodes on P7 adapter using *bcl2fastq v1.8.3* (Illumina) and a pair of 6 bp inline indices using a custom Perl script (https://doi.org/10.5061/dyrad.2j5b4). Trim_galore v0.4.1 (http://www.bioinformatics.babraham.ac.uk/projects/trim_galore/, accessed on June 24, 2016) wrapped in Cutadapt (Martin, 2011) was used to trim off adapter sequences and reads with low quality score (*Q* < 20). Read assembly was then performed following Yuan et al. (2016). PCR replicates were removed using a custom Perl script, and then the reads were parsed to each gene file according to their similarity to target sequences of the reference species, *Danio rerio* (https://doi.org/10.5061/dyrad.2j5b4). Trinity 2.2.0 (Grabherr et al., 2011) was used to perform initial read assembly with default parameters, and Geneious V7.1.5 (Kearse et al., 2012) was used to further merge assembled contigs. The assembled sequence that is most similar to the reference was selected according to Smith–Waterman algorithm (Smith & Waterman, 1981). Finally, the selected contigs were compared to the genome of the reference using blast v2.2.27 (Camacho et al., 2009). The selected sequences were considered orthologous only if they had the best hit in the target region of the reference genome. The final assembled sequences that passed the orthology checking were further filtered based on quality and completeness of the data. All loci were examined manually, and loci that had more than 90% missing data or uncorrectable segments in the alignment were excluded.

Consensus sequences were made for each target locus from assembled contigs using a custom Perl script (Yuan et al., 2016). Reads with adapter sequences trimmed and low‐quality reads excluded were mapped to the consensus sequences of each target using BWA v0.7.5 (Li & Durbin, 2009). The sequence map format (SAM) files were converted into binary format (BAM) using Samtools (Li et al., 2009). SNP sites were genotyped based on the BAM files using GATK‐3.2.2 (McKenna et al., 2010). GATK Best Practices recommendations were followed (DePristo et al., 2011; Van der Auwera et al., 2013). For most analyses, only one SNP per locus with the least amount of missing data and highest quality score was kept to meet the requirement of linkage equilibrium of those analyses. The SNP vcf file was converted into NEXUS file and STRUCTURE input file using custom Perl scripts (Yuan et al., 2016).

### 2.5 Genetic diversity and population clustering

An analysis of molecular variance (AMOVA) was performed on the SNP data using ARLEQUIN 3.5 with 10,000 permutations (Excoffier, Laval, & Schneider, 2005). The genetic variance was partitioned as among groups, that was among *C. nasus*, *C. nasus taihuensis*, and *C. brachygnathus*; among population within groups; and among individuals within population. Nucleotide diversity was estimated for each population using DnaSP (Rozas et al., 2017). Genetic distances among population were calculated using pairwise *F*_ST_ (Weir & Cockerham, 1984) implemented in ARLEQUIN3.5 based on the SNP data. A simple graphic representation of the *F*_ST_ values between populations was drawn using Rcmd implemented in ARLEQUIN3.5.

Cleaned sequences of all captured loci were concatenated to reconstruct a maximum likelihood tree to reveal relationships among the 138 individuals. The ML tree was reconstructed using RAxMLv8.0.0 under GTRGAMMA model with 1,000 bootstraps (Stamatakis, 2014). The resulting ML tree was visualized in Figtree v1.4.2 (http://tree.bio.ed.ac.uk/software/figtree/). Additionally, a median‐joining Network was created using Network 5.0.0.0 (Bandelt, Forster, & Rohl, 1999) to visualize genetic clustering of the 138 individuals. Network only uses polymorphic sites and there is a maximal limit for the number of characters that can be used in the analysis, so VCF file was filtered using Vcftools 0.1.15 (https://vcftools.github.io/), and only 808 SNP sites with less than 2% missing data were used for building the Network. VCF file was converted into *.arp file using PGDSpider 2.1.1.2 (Lischer & Excoffier, 2012), which was further edited and saved as input file (*.rdf) for Network analysis (Bandelt et al., 1999). Weight of characters was set as 10 for median‐joining (MJ) calculation, and weights of single‐nucleotide transversion and transition mutations were set as 1:1, epsilon = 0, frequency > 1, and MJ square option as active.

Genetic partitioning of the 138 individuals also was assessed using STRUCTURE v2.3.4 (Pritchard, Stephens, & Donnelly, 2000) based on the data containing one SNP per locus. The STRUCTURE runs were set with an initial burn‐in of 50,000 replicates, followed by 500,000 replicates for each *K* (number of genetic clusters). The analyses were run for *K* = 1–4, each replicated three times. The most likely number of K was determined using STRUCTURE HARVESTER 0.6.93 (Earl & VonHoldt, 2012).

Finally, principle coordinate analyses (PCA) was conducted using the ADE4 R packages (Jombart & Ahmed, 2011). PCA was computed with the R environment based on SNP data. Values of pc1 and pc2 were plotted to shown the genetic clustering of individuals from different populations.

### 2.6 Species delimitation

Three hypotheses based on previous morphological and molecular studies were compared. In the first scenario, *C. nasus*, *C. nasus taihuensis*, and *C. brachygnathus* were treated as one species. In the second model, *C. brachygnathus* was treated as a valid species and *C. nasus taihuensis* was not recognized as a separate subspecies of *C. nasus*. In the third model, all three taxa were considered valid. Additionally, a fourth model grouped *C. brachygnathus* and *C. nasus taihuensis* based on shared morphological traits.

The result of genetic clustering showed that individuals from DT, PY, and NY were close to each other, whereas individuals from CH, TH, and other lakes were clustered together, but there were also some individuals from NY and southern TH population showing intermediate genotypes probably due to hybridization. *Coilia brachygnathus* was originally described on a fish collected from DT (Kreyenberg & Pappenheim, 1908), and *C. nasus taihuensis* was described from TH and CH (Yuan et al., 1976). Therefore, we designated the samples of DT and PY as *C. brachygnathus* and fish from CH and eastern TH as *C. nasus taihuensis* for species delimitation to avoid mistakenly using admixed individuals.

Twenty‐two samples were randomly picked, such that each lake had 4–5 individuals: DT (5), PY (5), CH (4), TH (4), and SH (4). Two individuals of *C. mystus* were used as outgroup. Vcftools 0.1.15 was used to filter VCF files to exclude loci with more than 20% missing data. The resulting SNP sites were converted into NEXUS input file format for Bayes factor species delimitation (BFD*; Grummer, Bryson, & Reeder, 2014; Leaché, Fujita, Minin, & Bouckaert, 2014) using the SNAPP module in BEAST 2.3.2 (Bouckaert et al., 2014). Path sampling with 48 steps (100,000 MCMC steps, 10,000 pre‐burnin steps, alpha = 0.3) was conducted to estimate the marginal likelihood of each species delimitation model. Comparisons among candidate species models were performed using Bayes factors scale, 2ln(BF) (Kass & Raftery, 1995).

### 2.7 Testing hypotheses of freshwater invasion

**Figure 3:**
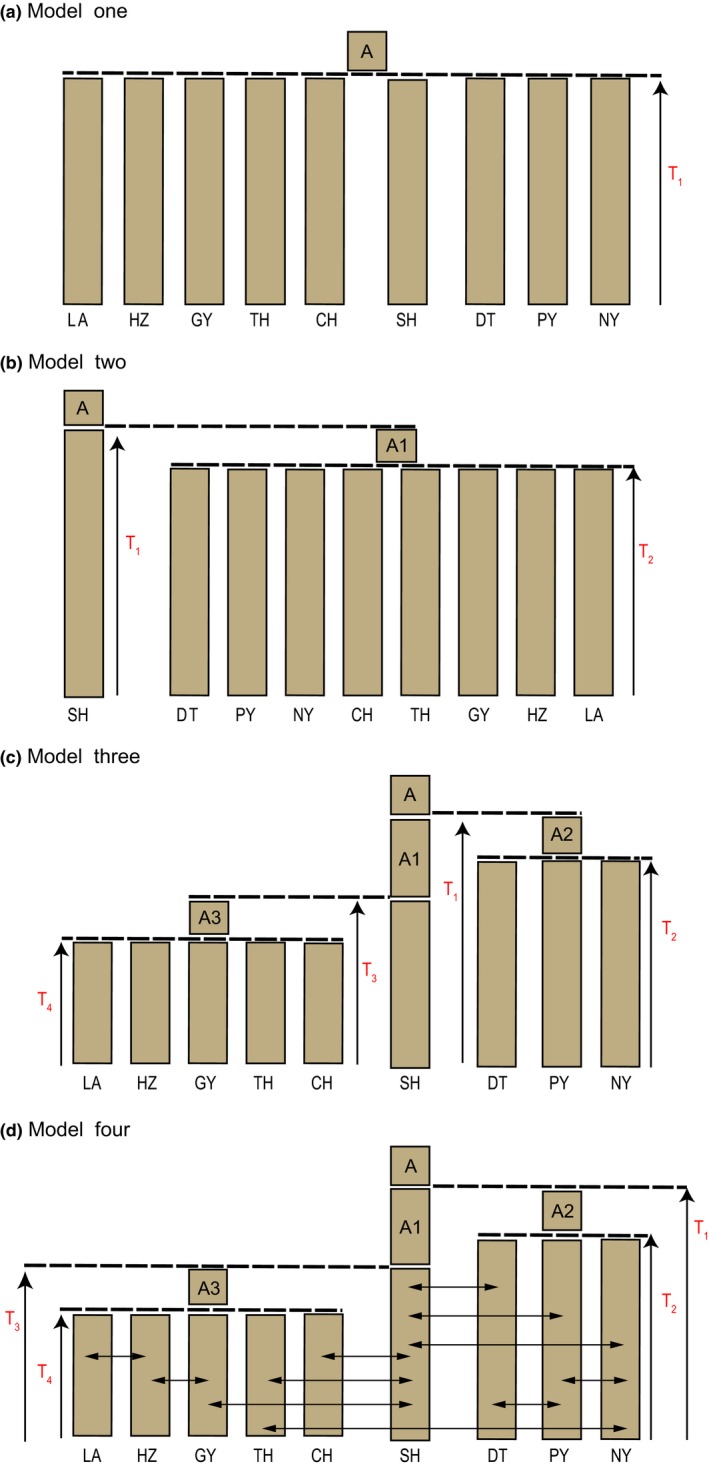
Hypotheses of history of freshwater invasion of *Coilia nasus*: (a) freshwater populations split from the anadromous population all at once; (b) all resident populations were derived from one common ancestral population, instead of splitting directly from the anadromous population as in model one; (c) two independent events of freshwater invasion: the populations of DT, PY, and NY are derived from the first freshwater invasion event and the other resident populations are the descendants of a second freshwater invasion event; (d) similar to model three but allowing migration between adjacent populations, indicated by arrows. A is the common ancestral population and other population names are abbreviated as in the text. T_1_, T_2_, T_3,_ and T_4_ are times of divergence (in generations)

Four models of freshwater invasion of the *C. nasus* species complex in the Yangtze River were tested (Figure 3). For model one, only one freshwater invasion event was hypothesized. Model two also had only one freshwater invasion event, but all resident populations were derived from a common ancestral population, instead of splitting directly from the anadromous population as in model one. In model three, two independent events of freshwater invasion were hypothesized based on the results of genetic clustering, so that the populations of DT, PY, and NY were derived from the first freshwater invasion event and the other resident populations were the descendants from the second freshwater invasion event. Model four was similar to model three but allowing migration between adjacent populations.

Fastsimcoal 2.5.2.21 was used to compare the different hypotheses (Excoffier, Dupanloup, Huerta‐Sanchez, Sousa, & Foll, 2013; Excoffier & Foll, 2011). Fastsimcoal2 can handle very complex evolutionary scenarios allowing for population resize, admixture events, and population fusion and fission, which can use the joint site frequency spectrum (JSFS) between populations as a summary statistics. VCF files of unlinked SNPs (one SNP per locus,) and linked SNPs (multiple SNPs per locus) were converted into *.arp files using PGDSpider 2.1.1.2, which were then used as input files of Alequin3.5 (Excoffier et al., 2005) for calculating folded joint SFS (*.obs).

Fastsimcoal simulation was based on folded (‐m) SFS by setting minimum (‐n) and a maximum (‐N) for 100,000 simulations, and minimum (‐l) and maximum (‐L) as 10 and 40 ECM cycles. A total of 100 independent runs were used to find optimized solutions for each model. The run that fits the observed JSFS best was used for model comparison using the Akaike information criterion (AIC). The template (*.tpl) files and parameter (*.est) files for all runs can be found in Dryad (https://doi.org/10.5061/dyrad.2j5b4).

Besides folded SFS, unfolded SFS also were calculated using *C. mystus* as an ancestral reference. Consensus sequences were made from assembled contigs of the five individuals of *C. mystus*. The consensus sequences were used as references for mapping trimmed reads of samples of *C. nasus* complex as described above. Unfolded joint SFS were calculated based on the mapped reads using ANGSD 0.918/0.919 (Korneliussen, Albrechtsen, & Nielsen, 2014). The unfolded SFS were used to compare the four models of freshwater invasion of the *C. nasus* species complex using fastsimcoal2 as described above, except for using d option for unfolded data.

Moreover, an approximate Bayesian computation (ABC; Beaumont, Zhang, & Balding, 2002) approach was used to estimate the parameters of the most supported model identified with the previous approaches. We built our simulations such that the simulated dataset had the same configuration (number of regions, sequence length, and sample sizes) as the observed data. We simulated 2,869 regions of 214 bp each using a mutation rate of 2.5 × 10^−8^ per site per generation (Excoffier et al., 2013). As summary statistics, the pairwise SFS were calculated. We generated 120,000 simulations using fastsimcoal2 v. 25,221 (Excoffier et al., 2013), and the demographic parameters were estimated from the 5,000 simulations closest to the observed dataset using both the neuralnet (Csillery, Francois, & Blum, 2012) and the rejection (Pritchard, Seielstad, Perez‐Lezaun, & Feldman, 1999) algorithm. Summary statistics have been reduced to 10 components using a partial least squares (PLS) regression implemented in the R library *mixomics* (Le Cao, Gonzalez, & Dejean, 2009). Analyses were performed in the R environment (R Core Team, 2014) with the library *abc* (Csillery et al., 2012).

### 2.8 Identifying outlier loci between different ecotypes

The SNP data were scanned for outlier loci between the anadromous populations and the resident populations using FDIST approach (Beaumont & Nichols, 1997) as implemented in ARLEQUIN35 (Excoffier et al., 2005), which uses coalescent simulations to get *p*‐values of locus‐specific *F*‐statistics (*F*_ST_) conditioned on observed levels of heterozygosities. Outlier loci show either significantly higher (divergent selection) or lower (balancing selection) *F*_ST_ compared to simulated neutral expectations. Based on the results of genetic clustering, the resident ecotype has two distinct groups: one including samples from Lake Dongting and Lake Poyang, and the other represented by fish from Lake Chao and Eastern Lake Tai. Thus, the anadromous population (SH) with fish from DT and PY, and the anadromous population (SH) with fish from CH and eastern TH were compared, respectively. Twenty thousand simulations were run with 100 demes per group and minimum and maximum expected heterozygosities from 0 and 1. The *F*_ST_ distribution was drawn using Rcmd in ARLEQUIN.

The outlier loci identified through the FDIST scanning were examined for the Gene Ontology Enrichment Analysis (GO analysis; Ashburner et al., 2000), which was performed using the Database for Annotation, Visualization and Integrated Discovery (DAVID, Version 6.8; https://david.ncifcrf.gov/), an online tool containing an integrated biological knowledgebase and analytic tools for systematically extracting biological meaning from a large number of genes. The GO analysis was performed on the disruptive loci only.

## 3 RESULTS

### 3.1 Habitat verification and sequencing results

The Sr/Ca ratio in center, margin, and middle position of the sagittal otoliths of the 32 fish collected from the lakes was stable, suggesting that they are resident individuals. On the contrary, the ratio of Sr/Ca of the anadromous fish showed a significant increased value in the middle and margin of the sagittal otolith (Table S1). Therefore, we confirmed that our lake samples are indeed from the resident populations.

There were 4,109,815 reads on average for each sample, after trimming off the adapter sequences and reads with low quality score (*Q* < 20). After removing the reads of PCR duplicates, 3,413,499 reads were kept. Read assembling produced 1,813 target loci for each sample on average, with the best sample had 2,397 loci and the worst one had 415 loci captured. The outgroup samples had 1,803 loci capture on average (Table S2). There were 3,858 loci that had sequence captured in at least one sample. All loci were examined manually, and 2,869 loci were kept after excluding the ones that had more than 90% missing data or uncorrectable segments in the alignment.

### 3.2 Genetic diversity and population structure

**Figure 4:**
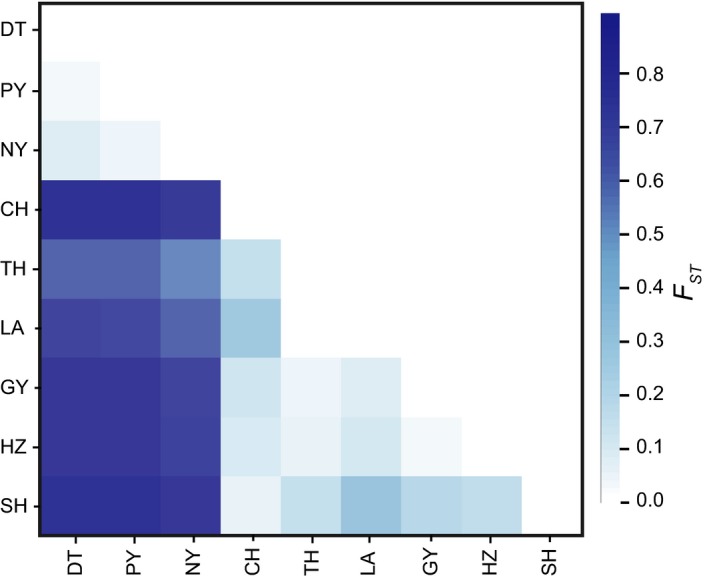
Matrix of pairwise *F*_ST_. The deeper color represents greater genetic distances

There were 2,513 loci that had at least one SNP site called. In total, 11,541 SNPs were called with an average of 4.6 SNPs per locus. Nucleotide diversity (Pi) ranged from 0.0011 in samples collected from Lake Poyang to 0.0029 in that of Lake Tai (Table S3). AMOVA showed that groups accounted for 50.68% variation, and populations within each group only contributed 3.47% variation. The variation between individuals within population was 45.58% (Table 2). Pairwise *F*_ST_ values were significant between all populations, except for between LA and TH, and between GY and HZ (Table S4). The pairwise *F*_ST_ values were high between the populations of DT, PY, and NY and other populations, but low between populations within each group, such as between DT and PY (Table S4; Figure 4).

**Table 2:** Results of analysis of molecular variance (AMOVA)

Source of variation	Sum of squares	Variance components	Percentage variation
Among groups: *C. nasus*, *C. nasus taihuensis*, *C. brachygnathus*	5,604	84.26	50.68
Among populations within groups	898	6.21	3.74
Within populations	7,398	75.78	45.58
Total	13,901	166.25	

**Figure 5:**
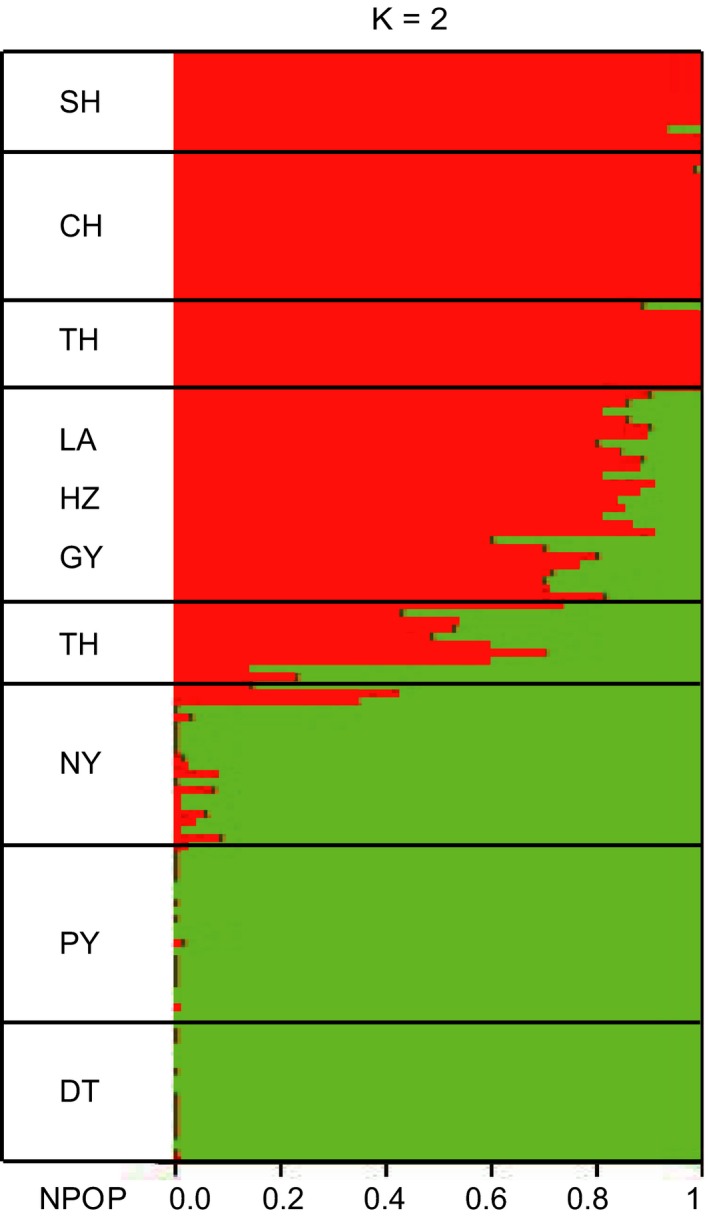
Structure analyses of nine populations using 2,513 SNP loci. Summary plot of estimates (*K* = 2). Each individual is represented by a single line, with *K* colored segments, and length proportional to each of the *K* inferred clusters. The NPOP (DT, PY…SH) correspond to the predefined populations

**Figure 6:**
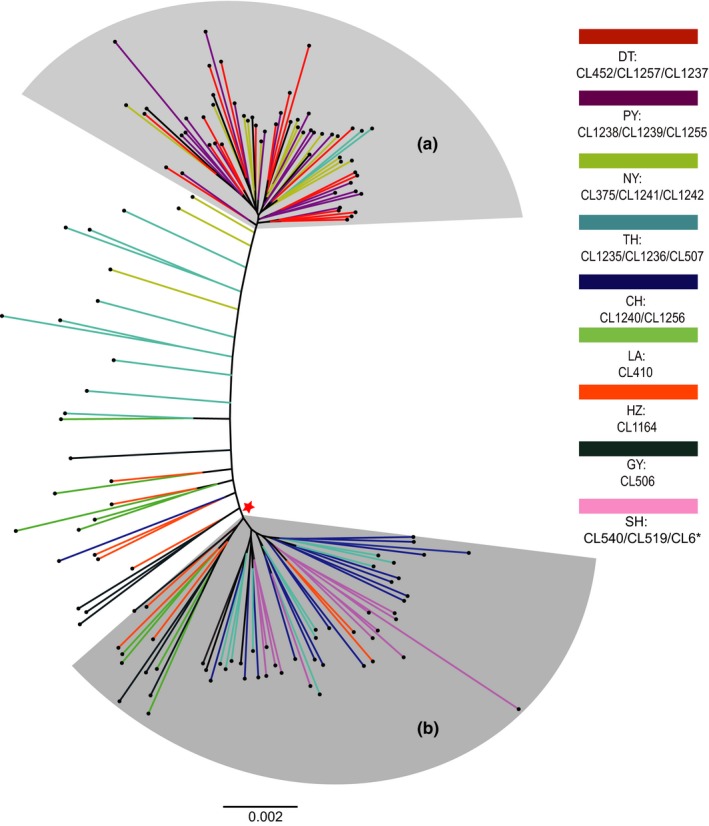
A ML tree based on sequences concatenating 2,869 loci (612,618 bp) reconstructed using RAxML under GTRGAMMAR model. (a) The clade of *C. brachygnathus*; and (b) the clade of *C. nasus taihuensis*/*C. nasus*. The star symbol indicates the root of the tree

Structure analysis supported two groups (*K* = 2) splitting DT, PY, and NY from the other six populations (Figure 5). NY, LA, GY, HZ, and southern and western TH had some admixture individuals (Figure 5). The 138 fish were largely clustered into two groups in the maximum likelihood tree built with RAxML. One group included DT, PY, and NY (Figure 6a), and the other group had SH, CH, GY, HZ, LA, and eastern TH (Figure 6b). Some fish from southern and western TH and NY were intermediate between the two clades (Figure 6). The results of the NETWORK analysis showed similar pattern with DT, PY, and NY forming a clade and the other populations forming another clade with some individuals from TH and NY located between the two clades (Figure S1). Finally, PCA revealed the same pattern, that is, the fish from DT, PY, and NY were clustered together and fish from other lakes were grouped with the anadromous fish (SH). Some individuals of NY and TH were mixed with each other (Figure S2).

### 3.3 Species delimitation on the *C. nasus* species complex

Excluding loci with more than 20% missing data resulted in 1,637 SNP sites for Bayes factor species delimitation (BFD*) analysis. The BFD* analysis supported the model splitting *C. brachygnathus* and *C. nasus* as different species, but further separating *C. nasus taihuensis* as a subspecies was not supported (BF = 2; Table 3 top). Grouping all populations as one species or separating them according to ecotypes were both rejected with a BF value of 9,430 and 8,570, respectively.

**Table 3:** Results of species delimitation based on 1,637 SNPs using Bayes factor species delimitation (BFD*)

Model	Taxon (sample locations)	No. species	Marginal likelihood	Rank	2ln(BF)
Model 1	*Coilia nasus* (DT, PY, CH, eastern TH, and SH)	1	−13,083	4	9,430
Model 2	*C. branchygnathus* (DT, PY)	2	−8,368	1	/
	*C. nasus* (CH, eastern TH, and SH)				
Model 3	*C. branchygnathus* (DT, PY)	3	−8,369	2	2
	*C. nasus taihuensis* (CH and eastern TH)				
	*C. nasus* (SH)				
Model 4	*C. branchygnathus* (DT, PY, CH, and eastern TH)	2	−12,653	3	8,570
	*C. nasus* (SH)				

Note

Only samples from type localities and no admixed samples were used. In model 1, *C. nasus*, *C. nasus taihuensis*, and *C. brachygnathus* were treated as one species, *C. nasus*. In model 2, *C. brachygnathus* was treated as a valid species and *C. nasus taihuensis* was not recognized as a separate subspecies of *C. nasus*. In model 3, all three taxa were considered valid. In model 4, *C. brachygnathus* and *C. nasus taihuensis* were grouped as one species based on their shared morphological traits.

### 3.4 The history of freshwater invasion of the *C. nasus* complex in the Yangtze River

Fastsimcoal2 analyses using folded SFS, calculated on both unlinked SNPs (one SNP per locus, 1,637 sites) and linked SNPs (multiple SNPs per locus, 7,316 sites), favored model two over model one (AIC 46,323 vs. 46,344 and 98,623 vs. 98,866), that is, the model including a common resident ancestor was preferred (Table 4). Two freshwater invasion events (model three) gained more support than one freshwater invasion event (model two; AIC 44,516 vs. 46,323 and 95,106 vs. 98,623). Fastsimcoal2 analyses using unfold SFS produced the same result, that is, two freshwater invasion events received higher support than one freshwater invasion (Table 4).

**Table 4:** Comparison on different models of freshwater invasion of *Coilia nasus* based on different site frequency spectrum (SFS) data

Model	SFS type^a^	SNPs^b^	MaxEstLhood	No. parameter	AIC^c^	Rank
Model one (All resident populations were derived directly from the anadromous population)	Folded	Unlinked	−23,162	10	46,344	4
	Folded	Linked	−49,423	10	98,866	4
	Unfolded	Unlinked	−1,130,375	10	2,260,769	4
Model two (The resident populations were derived from a common resident ancestor)	Folded	Unlinked	−23,150	11	46,323	3
	Folded	Linked	−49,301	11	98,623	3
	Unfolded	Unlinked	−1,128,404	11	2,256,830	3
Model three (Fresh water invasion occurred twice)	Folded	Unlinked	−22,245	13	44,516	2
	Folded	Linked	−47,540	13	95,106	2
	Unfolded	Unlinked	−1,128,208	13	2,256,442	2
Model four (Fresh water invasion occurred twice with migration allowed between adjacent populations)	Folded	Unlinked	−18,595	41	37,271	1
	Folded	Linked	−40,900	41	81,882	1
	Unfolded	Unli	−1,089,114	41	2,178,311	1

a SFS were calculated as folded and unfolded types.

b Unlinked means only one SNP site from each locus was used; linked means all SNP sites were used.

c AIC = 2*d* − 2ln(*L*).

**Figure 7:**
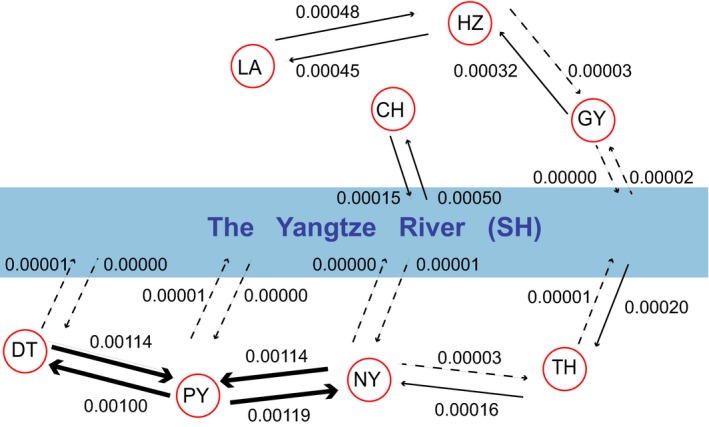
Estimated gene flow between adjacent populations: Lake Dongting (DT), Lake Poyang (PY), Lake Nanyi (NY), Lake Chao (CH), Lake Tai (TH), Luan (LA), Lake Hongze (HZ), Lake Gaoyou (GY), main channels, and estuary area of the Yangtze River (SH)

The model of two freshwater invasions with migration allowed between the adjacent populations (model four) received the highest support in all analyses (Table 4). The first freshwater invasion event happened around 4.07 Ma, and ancestral resident population then dispersed to Lake Dongting, Lake Poyang, and Lake Nanyi around 0.97 Ma (Table S5). The second freshwater invasion of *C. nasus* complex of the Yangtze River Basin occurred around 3.2 Ka, and the invaded population was distributed subsequently to the current Lake Chao, Lake Tai, and other freshwater lakes connected to the lower reaches of the Yangtze River around 3.1 Ka. These two most recent demographic events were also investigated using the ABC framework. The time of the second freshwater invasion was also estimated around 3.4 Ka (95% HPD 907–10,230 years ago), while the subsequent colonization of the current Lake Chao, Lake Tai, and the other connected lakes was estimated around 1.9 Ka (95 HPD 158–9,413 years ago; Table S6, Figure S3). High level of gene flow was also inferred between DT and PY, and between PY and NY (Figure 7). There was also noticeable gene flow between CH and SH, and from SH to TH, but the gene flow from TH to SH was low. Gene flow between SH and the other resident populations (DT, PY, NY, and GY) was negligible (Figure 7).

### 3.5 Genetic changes of invaded populations

We identified 124 outlier exons between the anadromous fish (SH) and the resident fish of DT and PY and 22 outliers between the anadromous fish (SH) and the resident fish of CH and eastern TH applying F‐DIST analysis (Table S7; Figure S4). Some loci of DT, PY, and SH had differentially fixed alternative alleles (*F*_ST_ = 1; Figure S4a). By contrast, *F*_ST_ between the resident fish of CH, eastern TH, and SH were lower with most values less than 0.4 (Figure S4b). From the 124 and 22 outliers found in the two comparisons, 120 and 21 outlier loci are disruptive (*F*_ST_ > 5% quantile). We found 9 loci were shared between the 120 and the 21 disruptive outliers identified from the two comparisons (Table S7).

We performed GO functional analyses using the 120, 21, and 9 loci separately. We obtained 25, 7, and 5 terms from those analyses (*p* < .05; Table S8; Figure S5). The terms with the most significant *p*‐values based on disruptive loci between *C. brachygnathus* and the anadromous *C. nasus* are WD40‐repeat and Armadillo‐type fold, whereas the most significant terms summarizing disruptive loci between the resident and anadromous *C. nasus* are transcription regulation and activator (Table S8; Figure S5).

## 4 DISCUSSION

### 4.1 Taxonomy of *Coilia* species complex

*Coilia brachygnathus* was distinguished from the other *Coilia* species by its short maxilla, not reaching to edge of gill cover (Whitehead et al., 1988), but later the same trait was found in *C. nasus taihuensis* and *C. nasus* collected from main channels of the Yangtze River (Ni & Wu, 2006; Tang et al., 2007). Moreover, both resident and anadromous ecotypes were confirmed coexisting in Lake Poyang based on different microchemistry patterns observed in their otoliths (Jiang, Zhou, Liu, Liu, & Yang, 2013). Thus, it was suspected that *C. brachygnathus* was not a valid species but an ecotype without genetic isolation from *C. nasus* (Tang et al., 2007). This hypothesis was supported by many subsequent molecular studies based on mitochondrial loci, showing that *C. brachygnathus* was mixed with *C. nasus* in the phylogenetic trees with low genetic distance between them (Cheng et al., 2011; Zhou et al., 2010).

The reason for nonmonophyletic mitochondrial sequences of *C. brachygnathus* might be an insufficient divergence time for complete lineage sorting between *C. brachygnathus* and *C. nasus*, which is a common phenomenon in many incipient species (Funk & Omland, 2003; Ross, 2014). Using many more independent loci and model‐based species delimitation methods should be better for elucidating species status and evolutionary history of *C. brachygnathus*. Indeed, we found a strong support for a valid species of *C. brachygnathus* separating from *C. nasus* using multilocus Bayes factor species delimitation (BFD*) with 1,637 SNPs (Table 3). The ML tree and Network also showed a clear clustering of *C. brachygnathus* distinct from the other *C. nasus* (Figure 6 and Figure S1). AMOVA showed that more than 50% of the variance was partitioned as among group difference, which was contributed mostly by difference between *C. brachygnathus* and *C. nasus* (Table 2). STRUCTURE analysis and PCA corroborated that *C. brachygnathus* and *C. nasus* formed two groups (Figure 5 and Figure S2). Therefore, we conclude that *C. brachygnathus* is a valid species.

*Coilia brachygnathus* completes its life cycle in freshwater in a handful lakes of the Yangtze River Basin, such as Lake Dongting and Lake Poyang, which are located in highly developed regions threatened heavily by anthropogenic activities. We recommend that conservation concerns of *C. brachygnathus* should call for more attention, since we confirm it as a different species from *C. nasus* and no gene exchange was detected between them. Our multilocus analyses produced a useful byproduct, that is, 68 loci were fixed between *C. brachygnathus* and the anadromous *C. nasus* (*F*_ST_ = 1; Table S7). Because no morphologic methods or molecular markers were available previously, methods such as measuring Sr/Ca ratio of the sagittal otolith was used to determine whether a fish were anadromous or resident (Jiang et al., 2016). The 68 nuclear loci that we identified here can readily be used to distinguish fish or fertilized eggs of the resident *C. brachygnathus* and the anadromous *C. nasus* occurring in the same lake, which is important in survey of spawning ground and conservation of both species (Jiang et al., 2016).

Morphological difference between the migratory and resident type of *C. nasus* might be the outcome of adaptive phenotypic variation to the freshwater habitat and diet. Our multilocus species delimitation did not support *C. nasus taihuensis* as a separate taxon, but grouped it with *C. nasus* (Table 3). ML tree, Network analysis, PCA, and STUCTURE analysis showed a similar pattern suggesting that fish from CH and TH are not separable from the anadromous fish (Figures 5 and 6; Figures S1 and S2). Palkovacs et al. (2008) also reported that landlocked alewife (*Alosa pseudoharengus*) populations had developed different morphological traits from the anadromous populations that were adapted to foraging on smaller prey items, while extensive haplotype sharing between the anadromous and landlocked populations was found. It is noteworthy that there are some admixed individuals found in Lake Tai and Lake Nanyi, which were nested in between the clades of *C. brachygnathus* and migratory *C. nasus* in the phylogenetic tree and the network (Figures 6 and S1). This may give an appearance that the landlocked ecotypes of *C. nasus* are hybrids of the two diverged species. However, there are two reasons suggesting that the landlocked *C. nasus* is not from a hybrid origin: (a) some populations of the landlocked *C. nasus* (all populations north of the Yangtze River) did not contact with *Coilia brachygnathus*, so those could not be resulted from hybridization; and (b) no admixed individuals were found in Lake Dongting and Lake Poyang, where *C. branchygnathus* and *C. nasus* contact each other directly. In conclusion, we confirmed that *C. brachygnathus* is a valid species, but we do not support *C. nasus taihuensis* as a valid subspecies of *C. nasus*, but an ecotype of *C. nasus* recently adapted to the freshwater landlocked environment.

### 4.2 Freshwater invasion of *Coilia* species

The paleo‐Yangtze drainage was fragmented and only the eastern part of it drained into the East China Sea (Wang, 1985). Chemical dating by electron microprobe provided solid evidence that draining the Yangtze River from the Tibetan plateau to the East China Sea should have formed before 2.58 Ma (Fan & Li, 2008; Fan et al., 2005), which is consistent with our estimation of the divergence time between *C. brachygnathus* and *C. nasus* (4.07 Ma). Thus, the middle Yangtze River must have joined the lower Yangtze River before the first freshwater invasion of *C. nasus* more than 4.07 Ma. Subsequently, lowered sea level caused drying up of most lakes in the middle‐lower reaches of the Yangtze River during glacial time, which might eradicate most lake‐resident fish and led to the isolation of *C. brachygnathus* from *C. nasus*. Modern lakes associated with the middle‐lower reach of the Yangtze River were not filled until around 6 Ka when the sea level rose up again (Yang et al., 2000). Research showed that the final formation of Lake Tai was about 3.7 Ka (Hong, 1991). Our estimates of the second freshwater invasion of *C. nasus* from different approaches are around 3.2 Ka and 3.4 Ka, consistent with the time of formation of lakes associated with the lower Yangtze River. Paleogeographic change often was the major cause of freshwater invasion of anadromous fishes (Van Nynatten, Bloom, Chang, & Lovejoy, 2015; Wilson et al., 2008). Our results revealed a case of multiple freshwater invasions driven by a series of complex paleographic events.

High level of gene flow was found between DT and PY, and between PY and NY (Figure 7), suggesting that *C. brachygnathus* in those lakes are connected somehow, and can be treated as one evolutionarily significant unit (ESU). The gene flow between anadromous *C. nasus* and *C. brachygnathus* is not significantly different from zero even though both species are found within the same lake (Jiang et al., 2016), but gene flow from resident *C. nasus* of TH to *C. brachygnathus* of NY is conspicuous. Different life history traits between *C. brachygnathus* and anadromous *C. nasus* and similar ecotype between *C. brachygnathus* and resident *C. nasus* may explain the pattern of gene flow described above. There are low but noticeable gene flows from the anadromous *C. nasus* to the resident *C. nasus* population, but the gene flows are much lower in reverse direction, suggesting that there must have been some anadromous fish strayed in lakes and reproduced with the resident individuals but few resident fish adventured into the sea for a migratory life style (Figure 7).

### 4.3 Genetic changes in landlocked ecotypes

Freshwater invaders may have evolved adaptively to cope with changes in osmoregulation and temperature fluctuation (Lee & Bell, 1999). For example, a population genomic study on parallel adaptation in three‐spined stickleback identified disruptive selection in candidate genes for development of osmoregulatory organs, and homeostasis of skeletal traits (Hohenlohe et al., 2010). Other common changes in fishes invading freshwater are traits related to foraging or water turbidity, such as positive selection in the rhodopsin of South American freshwater anchovies at sites known to be important for spectral tuning (Palkovacs et al., 2008; Van Nynatten et al., 2015). We identified 120 disruptive outliers by comparing *C. brachygnathus* to the anadromous population of *C. nasus*, and 21 disruptive outliers by comparing resident *C. nasus taihuensis* to the anadromous *C. nasus*. Nine outliers are found to be common between the two comparisons, indicating that independent freshwater invasion of *C. nasus* involved same mechanisms. Gene ontology analyses revealed that those 9 genes are related to regulation of both the transcription and translation (Table S8), suggesting that genetic changes in regulatory component might have played a central role in freshwater adaptation of *C. nasus*.

## CONFLICT OF INTEREST

None declared.

## AUTHOR CONTRIBUTIONS

C.L. and F.C. designed the project and wrote the initial manuscript. F.C. performed library prep and gene capture and analyzed data. P.M.D. did the ABC analyses. All authors contributed to editing and revising the manuscript.

## Data Availability

Gene capture data with adapters and low‐quality reads trimmed were deposited in GenBank (SRP131632). Custom Perl scripts, target sequences for baits designing, reference target sequences of *Danio rerio*, sequence alignments, and SNP data for all data analysis steps can be found in Dryad, entry https://doi.org/10.5061/dyrad.2j5b4.
