# Supplementary material for: Multiple freshwater invasions of the tapertail anchovy (Clupeiformes: Engraulidae) of the Yangtze River
Source: Ecol Evol. 2019 Oct 1;9(21):12202–15. doi: 10.1002/ece3.5708 (PMC6854117; doi:10.1002/ece3.5708)
Supplement: Supplementary file 1 [file ECE3-9-12202-s001.pdf]

Multiple freshwater invasions of the tapertail anchovy (Clupeiformes: Engraulidae) of the Yangtze River

Fangyuan Cheng[1,2,3], Qian Wang[1,2,3], Pierpaolo Maisano Delser[4,5], and Chenhong Li[1,2,3,*]

[1]Key Laboratory of Exploration and Utilization of Aquatic Genetic Resources, (Shanghai Ocean University), Ministry of Education, Shanghai 201306, China

[2]Shanghai Universities Key Laboratory of Marine Animal Taxonomy and Evolution, Shanghai 201306, China

[3]National Demonstration Center for Experimental Fisheries Science Education (Shanghai Ocean University), Shanghai 201306, China

[4]Department of Zoology, University of Cambridge, Cambridge CB2 3EJ, United Kingdom

[5] Smurfit Institute of Genetics, University of Dublin, Trinity College, Dublin 2, Ireland.

Table S1 Sr/Ca ratio of the sagittal otolith of 33 individuals from seven lakes (4-5 for each lake) and one fish from an anadromous population.

Collection site	Point of measurement*	Ca (ppm)	Sr (ppm)	1000 * Sr/Ca
The Yalu River	CL1176_2-01	396729.57	609.92	1.54
	CL1176_2-02	397097.81	302.39	0.76
	CL1176_2-03	395888.51	1333.25	3.37
Lake Dongting (DT)	CL452_1-01	396878.63	676.68	1.71
	CL452_1-02	396617.66	413.07	1.04
	CL452_1-03	397471.11	462.45	1.16
	CL452_9-01	396782.91	493.82	1.24
	CL452_9-02	396746.88	500.56	1.26
	CL452_9-03	390487.19	407.51	1.04
	CL452_4-01	397542.32	478.51	1.20
	CL452_4-02	397393.70	329.27	0.83
	CL452_4-03	391318.44	402.41	1.03
	CL1257_2-01	396939.29	715.43	1.80
	CL1257_2-02	396679.41	485.08	1.22
	CL1257_2-03	397161.17	415.00	1.04
	CL1257_3-01	397094.71	602.26	1.52
	CL1257_3-02	396168.78	659.74	1.67
	CL1257_3-03	397156.38	528.30	1.33
	CL1237_1-01	396968.51	734.38	1.85
	CL1237_1-02	397147.61	567.92	1.43
	CL1237_1-03	396891.42	731.77	1.84
	CL1237_3-01	396578.27	733.10	1.85
	CL1237_3-02	396841.98	708.36	1.78
	CL1237_3-03	396907.85	417.16	1.05
Lake Poyang (PY)	CL1255_1-01	396884.74	329.93	0.83
	CL1255_1-02	396921.35	266.74	0.67
	CL1255_1-03	397015.21	231.88	0.58
	CL1255_8-01	396291.01	369.94	0.93
	CL1255_8-02	396332.09	380.00	0.96
	CL1255_8-03	396584.56	258.30	0.65

Collection site	Point of measurement*	Ca (ppm)	Sr (ppm)	1000 * Sr/Ca
	CL1238_2-01	396343.65	814.89	2.06
	CL1238_2-02	396230.45	709.63	1.79
	CL1238_2-03	396842.67	499.48	1.26
	CL1238_3-01	396424.83	544.00	1.37
	CL1238_3-02	396031.14	650.51	1.64
	CL1238_3-03	397173.42	460.76	1.16
	CL1239_3-01	396175.77	497.02	1.25
	CL1239_3-02	396735.67	456.57	1.15
	CL1239_3-03	396603.15	405.49	1.02
	CL1239_2-01	396423.09	544.24	1.37
	CL1239_2-02	396701.51	480.29	1.21
	CL1239_2-03	396812.89	397.93	1.00
Lake Nanyi (NY)	CL375_3-01	397441.54	403.14	1.01
	CL375_3-02	396795.48	608.18	1.53
	CL375_3-03	366860.14	225.69	0.62
	CL1241_1-01	396280.64	530.03	1.34
	CL1241_1-02	397669.47	487.45	1.23
	CL1241_1-03	397579.55	548.88	1.38
	CL1241_3-01	396603.48	590.99	1.49
	CL1241_3-02	396664.13	551.29	1.39
	CL1241_3-03	397246.96	405.04	1.02
	CL1242_1-01	396917.97	529.37	1.33
	CL1242_1-02	396661.75	566.89	1.43
	CL1242_1-03	397637.38	464.85	1.17
	CL1242_7-01	397089.36	543.11	1.37
	CL1242_7-02	396696.81	415.95	1.05
	CL1242_7-03	397028.96	307.79	0.78
Lake Chao (CH)	CL1240_1-01	397419.28	371.63	0.94
	CL1240_1-02	397431.61	504.76	1.27
	CL1240_1-03	397002.52	755.27	1.90
	CL1240_4-01	396885.18	640.82	1.61
	CL1240_4-02	397365.07	544.07	1.37
	CL1240_4-03	396880.59	462.91	1.17

Collection site	Point of measurement*	Ca (ppm)	Sr (ppm)	1000 * Sr/Ca
	CL1256_9-01	396131.69	787.21	1.99
	CL1256_9-02	397463.54	640.30	1.61
	CL1256_9-03	397027.45	485.63	1.22
Lake Tai (TH)	CL507_4-01	397232.41	502.08	1.26
	CL507_4-02	397777.58	570.75	1.43
	CL507_4-03	396254.76	523.94	1.32
	CL507_9-01	396725.40	676.77	1.71
	CL507_9-02	397498.23	466.58	1.17
	CL507_9-03	392135.43	463.28	1.18
	CL1235_6-01	396567.75	501.28	1.26
	CL1235_6-02	397281.34	478.42	1.20
	CL1235_6-03	397419.39	548.07	1.38
	CL1235_3-01	396973.94	464.32	1.17
	CL1235_3-02	397299.21	344.69	0.87
	CL1235_3-03	397114.18	499.51	1.26
	CL1236_1-01	396847.26	483.56	1.22
	CL1236_1-02	397181.08	405.27	1.02
	CL1236_1-03	397346.74	489.75	1.23
	CL1236_7-01	396974.59	524.62	1.32
	CL1236_7-02	397068.56	380.53	0.96
	CL1236_7-03	397613.76	369.55	0.93
Lake Gaoyou (GY)	CL506_1-01	397063.03	854.93	2.15
	CL506_1-02	397138.23	747.90	1.88
	CL506_1-03	392838.89	654.97	1.67
	CL506_3-01	390554.33	785.04	2.01
	CL506_3-02	394034.30	622.62	1.58
	CL506_3-03	392209.74	655.65	1.67
	CL506_5-01	396704.77	845.59	2.13
	CL506_5-02	397263.68	814.02	2.05
	CL506_5-03	392739.93	695.08	1.77
Lake Hongze (HZ)	CL1164_5-01	396274.73	1107.13	2.79
	CL1164_5-02	396403.93	1102.45	2.78

Collection site	Point of measurement[*]	Ca (ppm)	Sr (ppm)	1000 * Sr/Ca
	CL1164_5-03	397624.09	477.91	1.20
	CL1164_8-01	396664.95	887.23	2.24
	CL1164_8-02	396475.56	862.87	2.18
	CL1164_8-03	397509.03	488.10	1.23

[*]01, center of the sagittal otoliths; 02, middle position of the sagittal otoliths; 03, margin of the sagittal otoliths

Table S2 Summary statistics of the sequencing results.

Sample ID	Locality	No. raw reads	No. filtered reads	No. captured loci
CL1164_1	Lake Hongze	4493540	3729480	1987
CL1164_10	Lake Hongze	5168818	4295348	1978
CL1164_2	Lake Hongze	9222906	7881688	2345
CL1164_3	Lake Hongze	6027054	4970082	2084
CL1164_4	Lake Hongze	9288124	8332648	2397
CL1164_5	Lake Hongze	6054860	4993412	2129
CL1164_6	Lake Hongze	7125322	5919942	2119
CL1164_7	Lake Hongze	6278520	5746530	2256
CL1164_8	Lake Hongze	2948468	2494902	1554
CL1164_9	Lake Hongze	3497244	2920554	1822
CL1235_1	Lake Tai	3625072	3034966	1845
CL1235_2	Lake Tai	3335050	2805944	1788
CL1235_3	Lake Tai	4513518	3782350	1918
CL1235_4	Lake Tai	4025478	3360556	1948
CL1235_5	Lake Tai	2252224	1876858	1642
CL1235_6	Lake Tai	7164480	5952176	2176
CL1236_1	Lake Tai	2508048	2082802	1754
CL1236_2	Lake Tai	3425690	2831710	1867
CL1236_3	Lake Tai	2712562	2252102	1825
CL1236_4	Lake Tai	2030634	1685384	1671
CL1236_5	Lake Tai	4697980	3898428	2015
CL1236_6	Lake Tai	2184896	1826336	1578
CL1236_7	Lake Tai	2093290	1740646	1719
CL1237_1	Lake Dongting	1832248	1550234	1273
CL1237_2	Lake Dongting	2654958	2222258	1664
CL1237_3	Lake Dongting	708962	599436	947
CL1237_4	Lake Dongting	3784106	3166098	1756
CL1237_5	Lake Dongting	2846004	2396202	1625
CL1237_6	Lake Dongting	4101252	3465196	1738
CL1238_1	Lake Poyang	1749090	1479936	1527
CL1238_2	Lake Poyang	2874110	2413864	1784
CL1238_3	Lake Poyang	2817690	2370956	1692
CL1238_6	Lake Poyang	6656226	5626420	2126
CL1238_7	Lake Poyang	2432140	2042770	1677
CL1239_1	Lake Poyang	5419018	4577952	1921
CL1239_2	Lake Poyang	2656334	2262660	1537
CL1239_3	Lake Poyang	3581568	3047146	1804
CL1239_4	Lake Poyang	3887358	3295230	1880

Sample ID	Locality	No. raw reads	No. filtered reads	No. captured loci
CL1239_5	Lake Poyang	4248686	3494708	1851
CL1239_6	Lake Poyang	4058252	3446440	1886
CL1239_7	Lake Poyang	3705010	3014578	1807
CL1240_1	Lake Chao	2596924	2107372	1591
CL1240_10	Lake Chao	7233778	5917128	2117
CL1240_2	Lake Chao	2645650	2226558	1681
CL1240_3	Lake Chao	5752056	4842362	1962
CL1240_4	Lake Chao	4127786	3489332	1764
CL1240_5	Lake Chao	2634138	2222120	1588
CL1240_6	Lake Chao	4460044	3763926	1915
CL1240_8	Lake Chao	3867202	3254466	1845
CL1240_9	Lake Chao	2612972	2190772	1719
CL1241_1	Lake Nanyi	3680688	3094544	1778
CL1241_2	Lake Nanyi	7810262	6391102	2148
CL1241_3	Lake Nanyi	4891230	4056206	1796
CL1241_4	Lake Nanyi	2071778	1719466	1623
CL1241_5	Lake Nanyi	3816440	3201050	1824
CL1241_6	Lake Nanyi	2823116	2360322	1603
CL1241_7	Lake Nanyi	2351162	1944506	1683
CL1242_1	Lake Nanyi	4894472	4119930	1783
CL1242_2	Lake Nanyi	3257062	2711926	1546
CL1242_3	Lake Nanyi	2751864	2338874	1756
CL1242_4	Lake Nanyi	3067446	2545486	1733
CL1242_5	Lake Nanyi	4142452	3457202	1905
CL1242_6	Lake Nanyi	3886942	3254132	1874
CL1242_7	Lake Nanyi	4546530	3808316	1858
CL1255_1	Lake Poyang	3669318	3092690	1576
CL1255_2	Lake Poyang	6577496	5913078	2111
CL1255_3	Lake Poyang	1886510	1586578	1311
CL1255_4	Lake Poyang	4922016	4138824	1780
CL1255_5	Lake Poyang	6387264	3951732	1910
CL1255_6	Lake Poyang	9201046	6456492	2115
CL1255_7	Lake Poyang	2970402	2414044	1697
CL1255_8	Lake Poyang	3619126	3043780	1467
CL1255_9	Lake Poyang	5064838	4203244	1823
CL1256_1	Lake Chao	2997140	2580526	1840
CL1256_10	Lake Chao	5656732	4779226	1999
CL1256_2	Lake Chao	4528318	3963832	2085
CL1256_3	Lake Chao	3494974	3002868	1923
CL1256_4	Lake Chao	4349726	3779328	2080

Sample ID	Locality	No. raw reads	No. filtered reads	No. captured loci
CL1256_5	Lake Chao	3367010	2841158	1789
CL1256_6	Lake Chao	3023814	2551002	1723
CL1256_7	Lake Chao	4541460	3979018	2052
CL1256_8	Lake Chao	4425050	3728888	1925
CL1256_9	Lake Chao	4435326	3725976	1961
CL1257_1	Lake Dongting	3097924	2620212	1571
CL1257_2	Lake Dongting	2081580	1746196	1209
CL1257_3	Lake Dongting	2308018	1935482	1425
CL1257_8	Lake Dongting	3322424	2729506	1417
CL375_1	Lake Nanyi	2677350	2190274	1692
CL375_2	Lake Nanyi	4691706	4078540	1967
CL375_3	Lake Nanyi	4540396	3775834	1891
CL375_4	Lake Nanyi	6667448	5658140	2137
CL375_5	Lake Nanyi	3643842	3049260	1870
CL410_1	Luan	7062704	5601394	2002
CL410_10	Luan	6367774	4422282	2035
CL410_3	Luan	5772676	4735708	1955
CL410_4	Luan	2044772	1491426	1381
CL410_5	Luan	2022552	1691392	1622
CL410_6	Luan	4713692	3875206	1914
CL410_7	Luan	5828714	4777118	2037
CL410_8	Luan	7691448	5370370	2083
CL410_9	Luan	3424242	2865968	1751
CL452_1	Lake Dongting	5171982	4332976	1948
CL452_2	Lake Dongting	2271898	1905754	1570
CL452_3	Lake Dongting	2415842	2021378	1627
CL452_4	Lake Dongting	2609336	2193878	1591
CL452_5	Lake Dongting	1699652	1426526	1434
CL452_6	Lake Dongting	2937088	2471592	1731
CL452_8	Lake Dongting	3839386	3222296	1734
CL452_9	Lake Dongting	2757480	2444396	1830
CL506_1	Lake Gaoyou	3785098	3125848	1829
CL506_10	Lake Gaoyou	4616238	3740820	1797
CL506_2	Lake Gaoyou	3570484	2914244	1692
CL506_3	Lake Gaoyou	2860642	2354120	1658
CL506_5	Lake Gaoyou	3529716	2902058	1744
CL506_7	Lake Gaoyou	4784470	4053848	1882
CL506_8_1	Lake Gaoyou	2103622	1998906	1815
CL506_9_1	Lake Gaoyou	2332080	2235400	1923
CL507_13	Lake Tai	2873548	2496448	1699

Sample ID	Locality	No. raw reads	No. filtered reads	No. captured loci
CL507_14	Lake Tai	5238088	4611786	2113
CL507_26	Lake Tai	9405418	8459310	2347
CL507_4	Lake Tai	6375198	5415788	2066
CL507_5	Lake Tai	3288592	2761784	1784
CL507_6	Lake Tai	2808600	2291900	1670
CL507_8	Lake Tai	5612868	4779692	2030
CL507_9	Lake Tai	2893060	2425278	1747
CL519_1	Port Luchao	5489608	4654770	2126
CL519_4	Port Luchao	3597350	2948574	1985
CL519_5	Port Luchao	3432790	2902434	1830
CL519_6	Port Luchao	6775072	5747948	2216
CL540_1	Chong-ming	5451762	4307284	2033
CL540_2	Chong-ming	4112856	3284644	1888
CL540_3	Chong-ming	5041974	4151124	1985
CL540_4	Chong-ming	6201018	5097172	1983
CL540_5	Chong-ming	3480354	2893624	1760
CL63	Port Luchao	4804034	4082188	1963
CL64_1	Jingjiang	4504378	2582714	415
CL66	Jingjiang	4203112	3447906	1913
CL68	Jingjiang	2594214	2125896	1708
Average		4109815	3413499	1813
**Outgroup (*Coilia mystus*)**				
CL81	Shanghai	4620416	3895516	1109
CL82	Shanghai	4610888	3893278	1196
CL83	Shanghai	17433766	15262342	2167
CL84	Shanghai	34760286	30370338	2270
CL447_7	Shanghai	27346970	23903514	2273
Average		13555840	11701326	1405

Table S3 Nucleotide diversity (Pi) of each population.

Population	No. fish	Theta (per site) from Pi
DT	18	0.00125
PY	21	0.00114
NY	19	0.0015
CH	19	0.00192
TH	21	0.00287
LA	9	0.0028
GY	8	0.00276
HZ	10	0.00247
SH	13	0.002

DT, Lake Dongting; PY, Lake Poyang; NY, Lake Nanyi; CH, Lake Chao; TH, Lake Tai; LA, Luan; GY, Lake Gaoyou; HZ, Lake Hongze; SH, Shanghai.

Table S4 Population pairwise F_{ST}.

	DT	PY	NY	CH	TH	LA	GY	HZ
DT								
PY	0.026*							
NY	0.071*	0.039*						
CH	0.730*	0.730*	0.679*					
TH	0.509*	0.510*	0.437*	0.131*				
LA	0.612*	0.610*	0.513*	0.243*	0.006			
GY	0.694*	0.692*	0.615*	0.108*	0.045*	0.072*		
HZ	0.697*	0.695*	0.623*	0.087*	0.056*	0.103*	0.035	
SH	0.751*	0.750*	0.695*	0.051*	0.131*	0.267*	0.169*	0.146*

$^{*}P < 0.05$

Table S5 Estimation of population parameters using fastsimcoal2 based on unlinked and folded site frequency spectrum (SFS).

Model four		
(Fresh water invasion occurred twice, MaxEstLhood = -18595)		
Parameter	Point estimation	Range[f]
N_{DT}[a]	130,273	130,235 – 140,786
N_{PY}	129,773	127,231 – 137,389
N_{NY}	130,690	130,576 – 139,619
N_{CH}	134,661	126,866 – 138,359
N_{TH}	130,970	126,253 – 140,285
N_{LA}	124,683	124,683 – 134,948
N_{GY}	120,568	120,568 – 140,114
N_{HZ}	80,967	80,967 – 92,168
N_{SH}	31,531	31,531 – 92,168
T_1[b]	3,128	1,316 – 5,128
T_2[c]	3,216	1,384 – 3,216
T_3[d]	97,050	13,646 – 274,004
T_4[e]	4,074,652	3,084,506 – 4,074,652

[a]N is the effective population size.

[b]T_1 is the time when a common resident population dispersed into the lakes of the lower reach of the Yangtze River (TH, CH, LA, HZ, GY). All time estimates were calculated as number of generations × 2 years, which is the average generation time.

[c]T_2 is the time when the anadromous population evolved a resident population ancestral to TH, CH, LA, HZ and GY.

[d]T_3 is the time when an ancestral resident population dispersed into Lake DT, PY and NY.

[e]T_4 is the time when the ancestral resident population of Lake DT, PY and NY formed.

[f]Range of estimates from 100 runs

Table S6 Parameter estimation under model four using the ABC framework. All time estimates and prior distributions are reported as number of generations × 2 years, which is the average generation time.

		Model four		
Parameter	Prior distribution	Median	Mode	95% HPD[c]
T_1[a]	U:20-10,000	1951	963	158 – 9413
T_2[b]	T_1 + U(20-2000)	3406	2578	907 – 10,230

[a]T_1 is the time when a common resident population dispersed into the lakes of the lower reach of the Yangtze River (TH, CH, LA, HZ, GY).
[b]T_2 is the time when the anadromous population evolved a resident population ancestral to TH, CH, LA, HZ and GY.
[c]Upper and lower limits of the 95% credible interval.

Table S7 Outliers indentified between *C. brachygnathus* (DT and PY) and the anadromous population of *C. nasus* (SH), and between the resident populations of *C. nasus* (CH and eastern TH) and the anadromous population of *C. nasus* (SH) respectively using F-DIST.

Gene name	Abbreviation	ENSEMBL gene ID	F_{ST}	*P*-value
Outliers indentified between DT, PY and SH:				
CREB/ATF bZIP transcription factor	CREBZF	ENSDARG00000089787	1	1.00E-07
vacuolar protein sorting 11	vps11	ENSDARG00000036338	1	1.00E-07
chromosome 21 open reading frame	C10H21orf59	ENSDARG00000035332	1	1.00E-07
mediator complex subunit 16	med16	ENSDARG00000040779	1	1.00E-07
si:ch211-233a24.2	si:ch211-233a24.2	ENSDARG00000062330	1	1.00E-07
phosphoinositide-3-kinase, regulatory subunit 4	pik3r4	ENSDARG00000060469	1	1.00E-07
zinc finger protein 407	ZNF407	ENSDARG00000087536	1	1.00E-07
serine/arginine repetitive matrix 1	srrm1	ENSDARG00000001244	1	1.00E-07
chromodomain helicase DNA binding protein 2	chd2	ENSDARG00000060687	1	1.00E-07
pyruvate dehydrogenase phosphatase regulatory subunit	PDPR	ENSDARG00000023113	1	1.00E-07
arginyl-tRNA synthetase	rars	ENSDARG00000054530	1	1.00E-07
TAF1 RNA polymerase II, TATA box binding protein (TBP)-associated factor 1	taf1	ENSDARG00000035330	1	1.00E-07
transmembrane protein 248	TMEM248	ENSDARG00000018825	1	1.00E-07
microfibrillar-associated protein 1	mfap1	ENSDARG00000018241	1	1.00E-07
\	\	ENSDARG00000077162	1	1.00E-07
ATP/GTP binding protein-like	AGBL4	ENSDARG00000096480	1	1.00E-07
RAP1 interacting factor homolog 1	rif1	ENSDARG00000062650	1	1.00E-07
splicing factor 3b, subunit 1	sf3b1	ENSDARG00000056138	1	1.00E-07
ribosomal protein 18	rpl8	ENSDARG00000014867	1	1.00E-07
nyctalopin	nyx	ENSDARG00000061791	1	1.00E-07

Gene name	Abbreviation	ENSEMBL gene ID	F_{ST}	*P*-value
adenylate cyclase 5	adcy5	ENSDARG00000091342	1	1.00E-07
T-box, brain 1b	tbr1b	ENSDARG00000004712	1	1.00E-07
G elongation factor, mitochondrial 1	gfm1	ENSDARG00000063624	1	1.00E-07
\	\	ENSDARG00000061952	1	1.00E-07
RNA binding motif protein 45	rbm45	ENSDARG00000063731	1	1.00E-07
leucyl-tRNA synthetase	larsb	ENSDARG00000019280	1	1.00E-07
CAP-GLY domain containing linker protein 2	clip2	ENSDARG00000059596	1	1.00E-07
cache domain containing 1	CACHD1	ENSDARG00000061590	1	1.00E-07
BRISC and BRCA1 A complex member 1	BABAM1	ENSGACG00000007777	1	1.00E-07
protein phosphatase 2, regulatory subunit B	ppp2r3c	ENSDARG00000043972	1	1.00E-07
proteasome (prosome, macropain) subunit, alpha type 3	psma3	ENSDARG00000086618	1	1.00E-07
vacuolar protein sorting 39 homolog	vps39	ENSDARG00000074471	1	1.00E-07
proteasome (prosome, macropain) 26S subunit, non- ATPase 14	psmd14	ENSDARG00000063100	1	1.00E-07
NADH dehydrogenase (ubiquinone) 1 alpha subcomplex	ndufa10	ENSDARG00000013333	1	1.00E-07
sodium leak channel	NALCN	ENSGACG00000001926	1	1.00E-07
cell division cycle 16 homolog	cdc16	ENSDARG00000055470	1	1.00E-07
ribulose-5-phosphate-3-epimerase	rpe	ENSDARG00000005251	1	1.00E-07
SET domain containing 2	SETD2	ENSGACG00000008671	1	1.00E-07
nucleotide-binding oligomerization domain	NOD1	ENSDARG00000036308	1	1.00E-07
myelin basic protein b	mbpb	ENSDARG00000089413	1	1.00E-07
glutamyl aminopeptidase	enpep	ENSDARG00000057064	1	1.00E-07
RAB interacting factor	rabif	ENSDARG00000002690	1	1.00E-07
GC-rich sequence DNA-binding factor	GCFC1	ENSORLG00000004845	1	1.00E-07

Gene name	Abbreviation	ENSEMBL gene ID	F_{ST}	*P*-value
WD repeat domain 67	wdr67	ENSDARG00000074623	1	1.00E-07
nucleotide-binding oligomerization domain containing 1	NOD1	ENSDARG00000036308	1	1.00E-07
translin	tsn	ENSDARG00000041830	1	1.00E-07
sec1 family domain containing 1	SCFD1	ENSORLG00000010536	1	1.00E-07
poly (ADP-ribose) polymerase family, member 1	parp1	ENSDARG00000019529	1	1.00E-07
spastic paraplegia 7	SPG7	ENSDARG00000068187	1	1.00E-07
DEAD (Asp-Glu-Ala-Asp) box polypeptide 59	ddx59	ENSDARG00000022177	1	1.00E-07
FtsJ homolog 3	FTSJ3	ENSORLG00000005910	1	1.00E-07
\	\	ENSTNIG00000019262	1	1.00E-07
vacuolar protein sorting 39 homolog	vps39	ENSDARG00000074471	1	1.00E-07
si:ch211-233a24.2	si:ch211-233a24.2	ENSDARG00000062330	1	1.00E-07
mitochondrial ribosomal protein L21	MRPL21	ENSTNIG00000006295	1	1.00E-07
\	\	ENSDARG00000061952	1	1.00E-07
transmembrane and tetratricopeptide repeat containing 4	tmtc4	ENSDARG00000020447	1	1.00E-07
thioredoxin domain containing 9	TXNDC9	ENSTNIG00000013871	1	1.00E-07
Sp3 transcription factor	SP3	ENSTNIG00000016947	1	1.00E-07
CWC22 spliceosome-associated protein homolog	cwc22	ENSDARG00000014008	1	1.00E-07
phosphatidylinositol glycan anchor biosynthesis, class O	pigo	ENSDARG00000011743	1	1.00E-07
kinesin family member C3	KIFC3	ENSTNIG00000002550	1	1.00E-07
zinc finger protein 236	znf236	ENSDARG00000095890	1	1.00E-07
ubiquitin protein ligase E3 component n-recognin 5	ubr5	ENSDARG00000018192	1	1.00E-07
electron-transfer-flavoprotein, beta polypeptide	etfb	ENSDARG00000009250	1	1.00E-07
rabaptin, RAB GTPase binding effector protein 1	rabep1	ENSDARG00000059600	1	1.00E-07

Gene name	Abbreviation	ENSEMBL gene ID	F_{ST}	*P*-value
glutamyl aminopeptidase	enpep	ENSDARG00000057064	1	1.00E-07
\	\	ENSTNIG00000019381	1	1.00E-07
EF-hand calcium binding domain 7	efcab7	ENSDARG00000020279	0.38502	0.000218033
WD repeat domain 6	WDR6	ENSGACG00000005768	0.38502	0.000218033
YLP motif containing 1	ylpm1	ENSDARG00000043680	0.38502	0.000218033
UPF2 regulator of nonsense transcripts homolog	upf2	ENSDARG00000074658	0.38502	0.000218033
eukaryotic elongation factor, selenocysteine-tRNA- specific	eefsec	ENSDARG00000004696	0.38502	0.000218033
jumonji domain containing 7	jmjd7	ENSDARG00000035546	0.38502	0.000218033
EF-hand calcium binding domain 7	EFCAB7	ENSTNIG00000018103	0.38502	0.000218033
dynein, axonemal, heavy chain 2	DNAH2	ENSDARG00000087352	0.379592	0.000944453
dynein, cytoplasmic 2, heavy chain 1	DYNC2H1	ENSGACG00000008068	0.379592	0.000944453
WD repeat domain 81	WDR81	ENSDARG00000079702	0.567753	0.001148224
integrator complex subunit 9	ints9	ENSDARG00000067913	0.261364	0.002336613
ubiquitin specific peptidase 47	USP47	ENSGACG00000015728	0.261364	0.002336613
baculoviral IAP repeat-containing 6	birc6	ENSDARG00000074166	0.261364	0.002336613
\	\	ENSGACG00000002639	0.261364	0.002336613
CCR4-NOT transcription complex, subunit 1	cnot1	ENSDARG00000004174	0.261364	0.002336613
phosphoinositide-3-kinase, regulatory subunit 4	pik3r4	ENSDARG00000060469	0.261364	0.002336613
metal-regulatory transcription factor 1	mtf1	ENSDARG00000090180	0.261364	0.002336613
si:ch73-196k8.2	si:ch73-196k8.2	ENSDARG00000077431	0.261364	0.002336613
DnaJ (Hsp40) homolog, subfamily C, member 3	dnajc3	ENSDARG00000041110	0.261364	0.002336613
peroxisomal membrane protein 4, 24kDa	PXMP4	ENSDARG00000059555	0.261364	0.002336613
integrator complex subunit 1	ints1	ENSDARG00000059631	0.261364	0.002336613

Gene name	Abbreviation	ENSEMBL gene ID	F_{ST}	*P*-value
golgin A4	golga4	ENSDARG00000075331	0.474453	0.003391352
nuclear receptor interacting protein 1	NRIP1	ENSGACG00000020154	0.37406	0.003507723
CWC22 spliceosome-associated protein homolog	cwc22	ENSDARG00000014008	0.256603	0.006488134
PMS2 postmeiotic segregation increased 2	PMS2	ENSTNIG00000012880	0.256603	0.006488134
propionyl Coenzyme A carboxylase, beta polypeptide	pccb	ENSDARG00000038910	0.130528	0.010682434
cyclin D binding myb-like transcription factor 1	dmtf1	ENSDARG00000025824	0.130528	0.010682434
nitrogen permease regulator-like 3	nprl3	ENSDARG00000010918	-0.05882	0.011050213
coiled-coil domain containing 130	CCDC130	ENSGACG00000019822	0.468523	0.011606253
Leo1, Paf1/RNA polymerase II complex component, homolog	leo1	ENSDARG00000055357	0.123007	0.013120661
phosphatidylinositol glycan, class K	pigk	ENSDARG00000024479	0.123007	0.013120661
mediator complex subunit 7	med7	ENSDARG00000016318	0.251769	0.0160146
microtubule associated serine/threonine kinase-like	MASTL	ENSGACG00000005087	0.251769	0.0160146
MON2 homolog	MON2	ENSORLG00000002173	0.251769	0.0160146
golgi autoantigen, golgin subfamily a, 3	golga3	ENSDARG00000062511	0.251769	0.0160146
ubiquitin protein ligase E3 component n-recognin 4	ubr4	ENSDARG00000009549	0.251769	0.0160146
transformation/transcription domain-associated protein	TRRAP	ENSDARG00000090442	-0.04762	0.025280462
24-dehydrocholesterol reductase	dhcr24	ENSDARG00000013236	-0.03392	0.029177708
SEC23 interacting protein	sec23ip	ENSDARG00000061413	0.11592	0.033654472
COP9 constitutive photomorphogenic homolog subunit 5	cops5	ENSDARG00000057624	0.11592	0.033654472
Uncharacterized protein	CABZ01076938.1	ENSDARG00000007398	0.11592	0.033654472
ubiquitin specific peptidase 47	USP47	ENSGACG00000015728	0.11592	0.033654472
serine hydroxymethyltransferase 1	SHMT1	ENSGACG00000007764	0.11592	0.033654472
NOP14 nucleolar protein homolog	NOP14	ENSGACG00000016531	0.11592	0.033654472

Gene name	Abbreviation	ENSEMBL gene ID	F_{ST}	*P*-value
solute carrier family 15, member 5	SLC15A5	ENSDARG00000089974	0.11592	0.033654472
nucleoporin 155	nup155	ENSDARG00000001777	0.11592	0.033654472
zinc finger CCCH-type containing 18	zc3h18	ENSDARG00000062506	0.11592	0.033654472
tubulin, gamma 1	tubg1	ENSDARG00000015610	0.11592	0.033654472
MAP-kinase activating death domain	MADD	ENSTNIG00000009591	0.11592	0.033654472
HECT, UBA and WWE domain containing 1, E3 ubiquitin protein ligase	HUWE1	ENSTNIG00000015085	0.11592	0.033654472
kinase non-catalytic C-lobe domain (KIND) containing 1	KNDC1	ENSGACG00000002854	0.246862	0.035067262
protein O-fucosyltransferase 1	pofut1	ENSDARG00000008953	0.246862	0.035067262
nucleoporin 205	nup205	ENSDARG00000042530	0.335545	0.041133849
\	\	ENSDARG00000030945	0.461538	0.041961314
laminin, beta 2	LAMB2	ENSTNIG00000002532	0.602186	0.04460292
GTPase activating Rap/RanGAP domain-like 3	GARNL3	ENSDARG00000060631	-0.0323	0.046711224
Outliers indentified between CH, eastern TH and SH:				
pantothenate kinase 4	pank4	ENSDARG00000040238	-0.05757	1.99E-57
Wilms tumor 1 associated protein	wtap	ENSDARG00000042642	0.751438	1.51E-34
dynein, axonemal, heavy chain 2	DNAH2	ENSDARG00000087352	0.312102	0.000147355
UPF2 regulator of nonsense transcripts homolog	upf2	ENSDARG00000074658	0.312102	0.000147355
GCN1 general control of amino-acid synthesis 1-like 1	gcn1l1	ENSDARG00000058419	0.502619	0.000710123
eukaryotic translation initiation factor 2B, subunit 4	eif2b4	ENSDARG00000014004	0.69526	0.000731825
ATP-binding cassette, sub-family B (MDR/TAP), member 7	abcb7	ENSDARG00000062795	0.576795	0.00278513
synovial apoptosis inhibitor 1, synoviolin	syvn1	ENSDARG00000017842	0.198813	0.003154525
baculoviral IAP repeat-containing 6	birc6	ENSDARG00000074166	0.198813	0.003154525

Gene name	Abbreviation	ENSEMBL gene ID	F_{ST}	*P*-value
CCR4-NOT transcription complex, subunit 1	cnot1	ENSDARG00000004174	0.198813	0.003154525
metal-regulatory transcription factor 1	mtf1	ENSDARG00000090180	0.198813	0.003154525
cyclin D binding myb-like transcription factor 1	dmtf1	ENSDARG00000025824	0.086262	0.007330681
mediator complex subunit 7	med7	ENSDARG00000016318	0.192964	0.009123008
protein O-fucosyltransferase 1	pofut1	ENSDARG00000008953	0.192964	0.009123008
recombination activating gene 2	rag2	ENSDARG00000052121	0.23057	0.024372972
NOL1/NOP2/Sun domain family, member 2	nsun2	ENSDARG00000056665	0.26306	0.024685287
laminin, beta 2	LAMB2	ENSTNIG00000002532	0.16733	0.031588709
Leo1, Paf1/RNA polymerase II complex component, homolog	leo1	ENSDARG00000055357	0.077246	0.033827972
claspin	CLSPN	ENSTNIG00000006330	0.239412	0.042230094
mediator complex subunit 23	med23	ENSDARG00000029157	0.276139	0.045544153
spondin 2b, extracellular matrix protein	spon2b	ENSDARG00000002732	0.276139	0.045544153
cleavage and polyadenylation specific factor 3-like	cpsf3l	ENSDARG00000025212	0.229144	0.045927281

*Genes underlined are common ones found in both comparison

Table S8 Results from Gene Ontology Enrichment Analysis (GO analysis; Current Background, *Danio rerio*).

Category	Numbrs	Term	Count	%	*P*-Value
Category found comparing DT, PY and SH:					
GOTERM_BP_DIRECT	GO:0030318	melanocyte differentiation	3	3.75	0.005247
GOTERM_BP_DIRECT	GO:0006412	translation	5	6.25	0.026756
GOTERM_BP_DIRECT	GO:0006355	regulation of transcription, DNA-templated	9	11.25	0.097841
GOTERM_BP_DIRECT	GO:0006414	translational elongation	2	2.50	0.099951
GOTERM_CC_DIRECT	GO:0005643	nuclear pore	3	3.75	0.009044
GOTERM_CC_DIRECT	GO:0044611	nuclear pore inner ring	2	2.50	0.011007
GOTERM_CC_DIRECT	GO:0030897	HOPS complex	2	2.50	0.018279
GOTERM_CC_DIRECT	GO:0005813	centrosome	3	3.75	0.048728
GOTERM_MF_DIRECT	GO:0016874	ligase activity	4	5.00	0.017843
GOTERM_MF_DIRECT	GO:0017056	structural constituent of nuclear pore	2	2.50	0.04513
GOTERM_MF_DIRECT	GO:0008237	metallopeptidase activity	3	3.75	0.067351
GOTERM_MF_DIRECT	GO:0003924	GTPase activity	3	3.75	0.088321
GOTERM_MF_DIRECT	GO:0003746	translation elongation factor activity	2	2.50	0.091267
INTERPRO	IPR017986	WD40-repeat-containing domain	9	11.25	8.19E-06
INTERPRO	IPR016024	Armadillo-type fold	7	8.75	9.87E-04
INTERPRO	IPR015943	WD40/YVTN repeat-like-containing domain	7	8.75	0.00139
INTERPRO	IPR001680	WD40 repeat	5	6.25	0.014067
INTERPRO	IPR014729	Rossmann-like alpha/beta/alpha sandwich fold	3	3.75	0.014788
INTERPRO	IPR003126	Zinc finger, N-recognin	2	2.50	0.023756
INTERPRO	IPR000547	Clathrin, heavy chain/VPS, 7-fold repeat	2	2.50	0.030441

Category	Numbrs	Term	Count	%	*P*-Value
INTERPRO	IPR009080	Aminoacyl-tRNA synthetase, class 1a, anticodon-binding	2	2.50	0.043675
INTERPRO	IPR011990	Tetratricopeptide-like helical	4	5.00	0.044085
INTERPRO	IPR027417	P-loop containing nucleoside triphosphate hydrolase	9	11.25	0.046676
INTERPRO	IPR000555	JAB1/Mov34/MPN/PAD-1	2	2.50	0.046956
INTERPRO	IPR013026	Tetratricopeptide repeat-containing domain	3	3.75	0.075404
INTERPRO	IPR019734	Tetratricopeptide repeat	3	3.75	0.075404
INTERPRO	IPR000795	Elongation factor, GTP-binding domain	2	2.50	0.085468
SMART	SM00320	WD40	5	6.25	0.013569
SMART	SM00396	ZnF_UBR1	2	2.50	0.02394
SMART	SM00232	JAB_MPN	2	2.50	0.044014
SMART	SM00028	TPR	3	3.75	0.062548
UP_KEYWORDS	/	Coiled coil	21	26.25	0.003672
UP_KEYWORDS	/	Protein biosynthesis	3	3.75	0.044843
UP_KEYWORDS	/	Nucleus	11	13.75	0.060101
UP_KEYWORDS	/	Elongation factor	2	2.50	0.073996
UP_KEYWORDS	/	Activator	3	3.75	0.088515
UP_SEQ_FEATURE	/	compositionally biased region:Poly-Phe	2	2.50	0.016182
Category found comparing CH, eastern TH and SH:					
GOTERM_BP_DIRECT	GO:0006351	transcription, DNA-templated	8	47.06	9.55E-05
GOTERM_BP_DIRECT	GO:0006355	regulation of transcription, DNA-templated	4	23.53	1.50E-04
GOTERM_BP_DIRECT	GO:0006417	regulation of translation	5	29.41	8.80E-04
GOTERM_CC_DIRECT	GO:0005634	nucleus	5	29.41	0.001141
SMART	SM00382	AAA	9	52.94	0.001866

Category	Numbrs	Term	Count	%	*P*-Value
UP_KEYWORDS	/	Nucleus	5	29.41	0.004178
UP_KEYWORDS	/	Activator	5	29.41	0.029023
UP_KEYWORDS	/	Transcription regulation	2	11.76	0.037828
UP_KEYWORDS	/	Transcription	2	11.76	0.08646
UP_KEYWORDS	/	Cell cycle	2	11.76	0.094476
Category found based on common loci in both comparison:					
GOTERM_BP_DIRECT	GO:0006351	transcription, DNA-templated	3	50.00	0.014054
GOTERM_BP_DIRECT	GO:0006355	regulation of transcription, DNA-templated	3	50.00	0.041038
UP_KEYWORDS	/	Transcription regulation	3	50.00	0.007546
UP_KEYWORDS	/	Transcription	3	50.00	0.008642
UP_KEYWORDS	/	Activator	2	33.33	0.032697
UP_KEYWORDS	/	Nucleus	3	50.00	0.050361

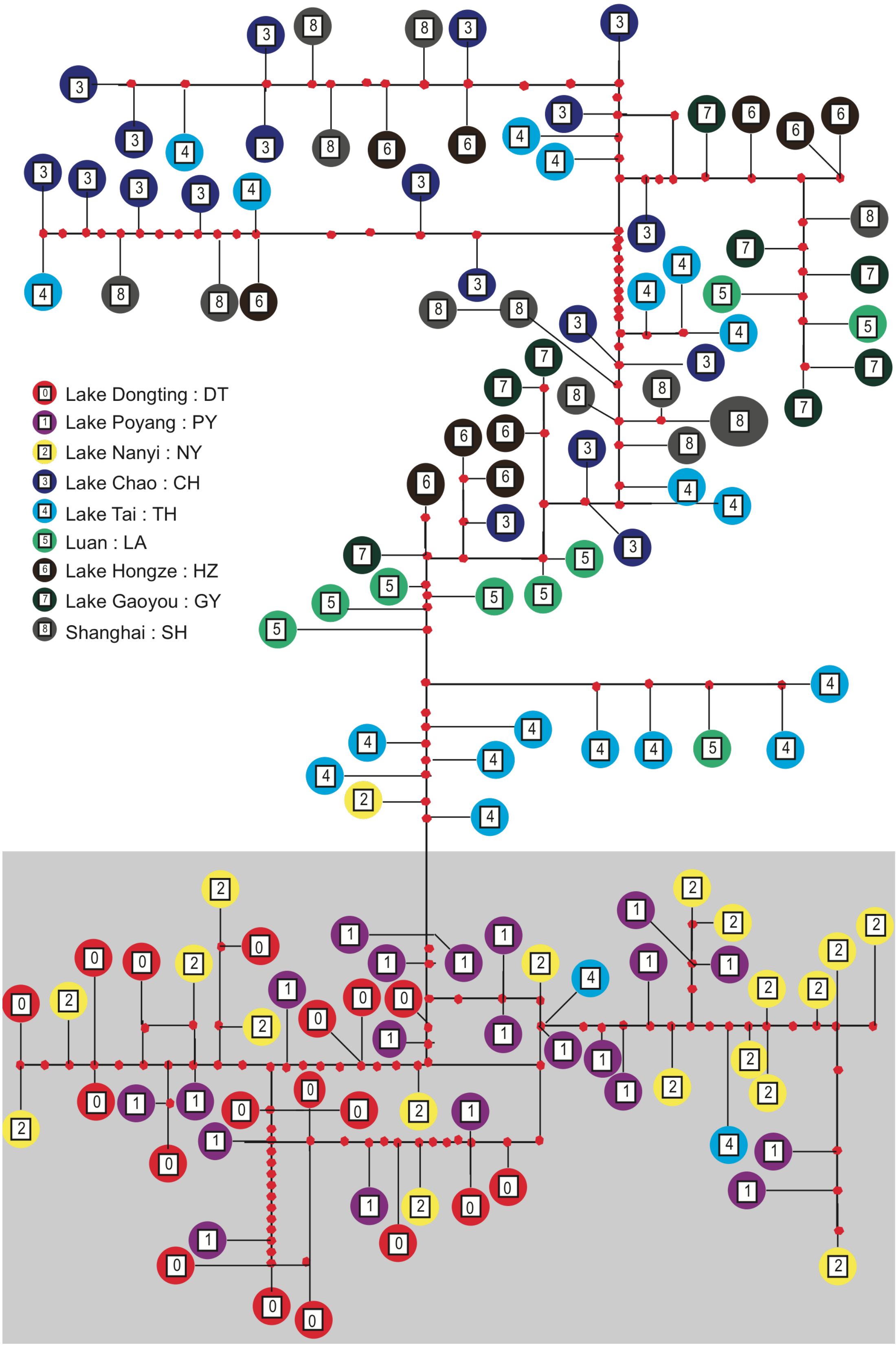

Fig. S1 A network of 138 individuals based on 808 SNPs loci. The dots between the nodes indicate the number of substitutions. The area of each circle is proportional to the corresponding haplotypes.

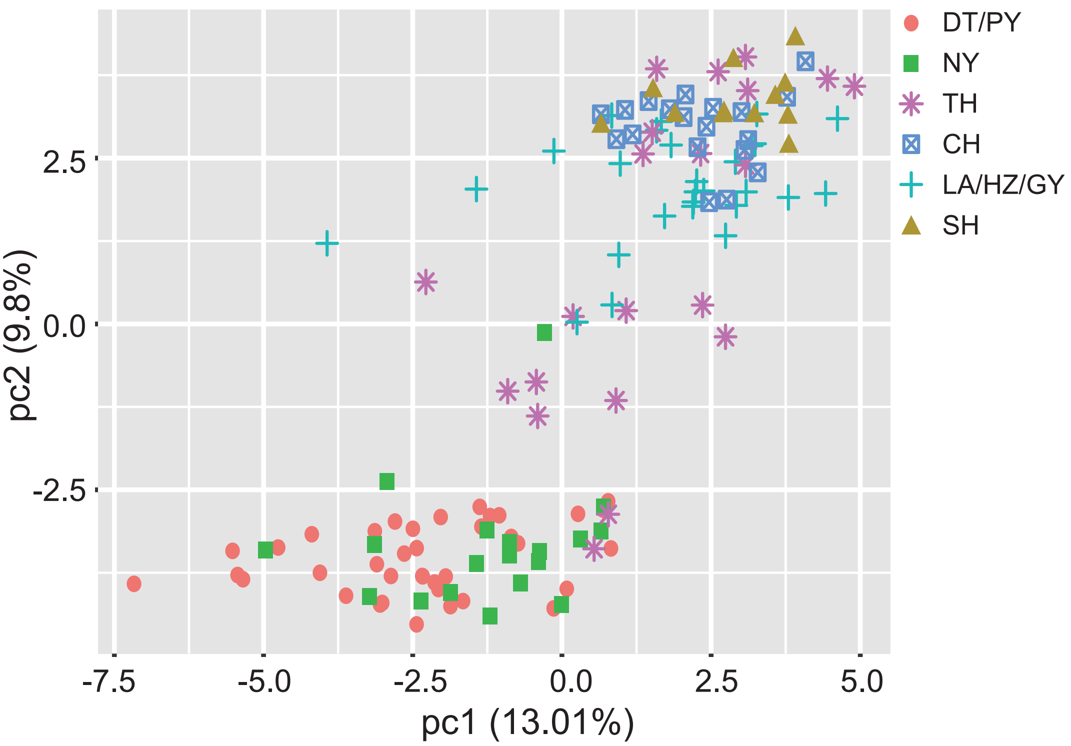

Fig. S2 Plot of the pc1 and pc2 components of a PCA analysis on 2,513 polymorphic SNP markers of the nine populations: Lake Dongting (DT), Lake Poyang (PY), Lake Nanyi (NY), Lake Chao (CH), Lake Tai (TH), Luan (LA), Lake Hongze (HZ), Lake Gaoyou (GY) and the Yangtze River (SH). Each point represents an individual colored according to different populations.

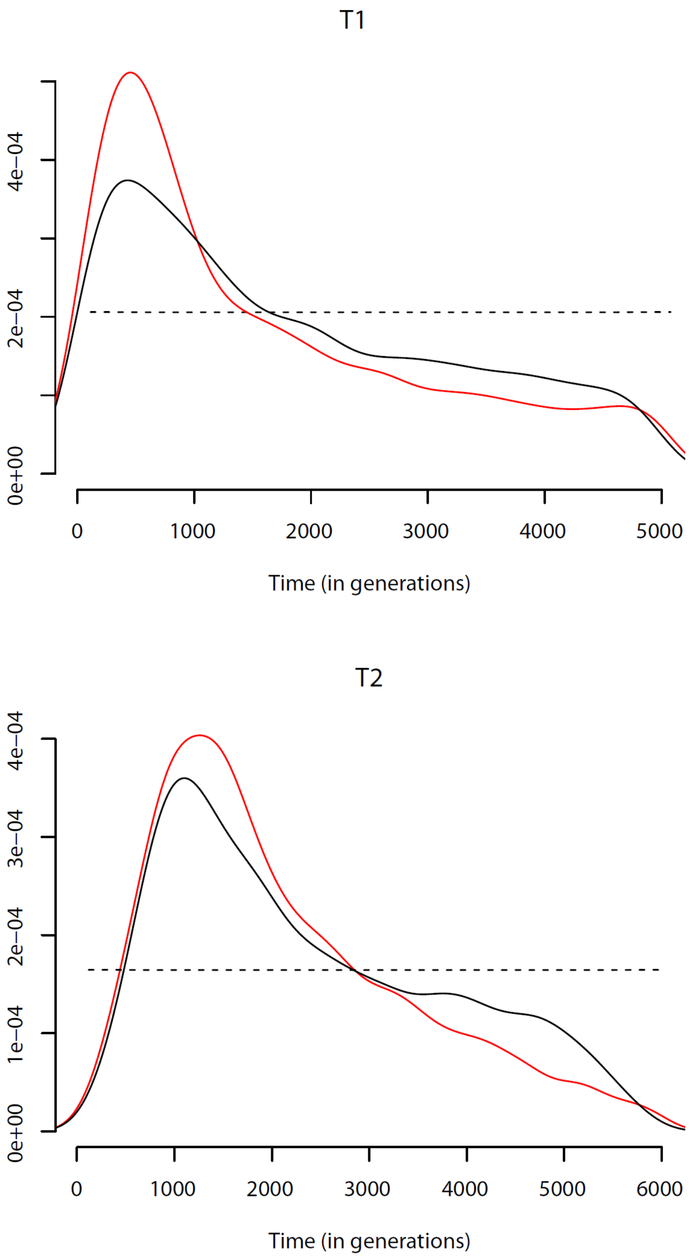

Fig. S3 Posterior distributions of T1 and T2 using the ABC framework. Prior distributions are represented by the dotted line while rejection and neuralnet algorithm are represented by the solid black and red line respectively.

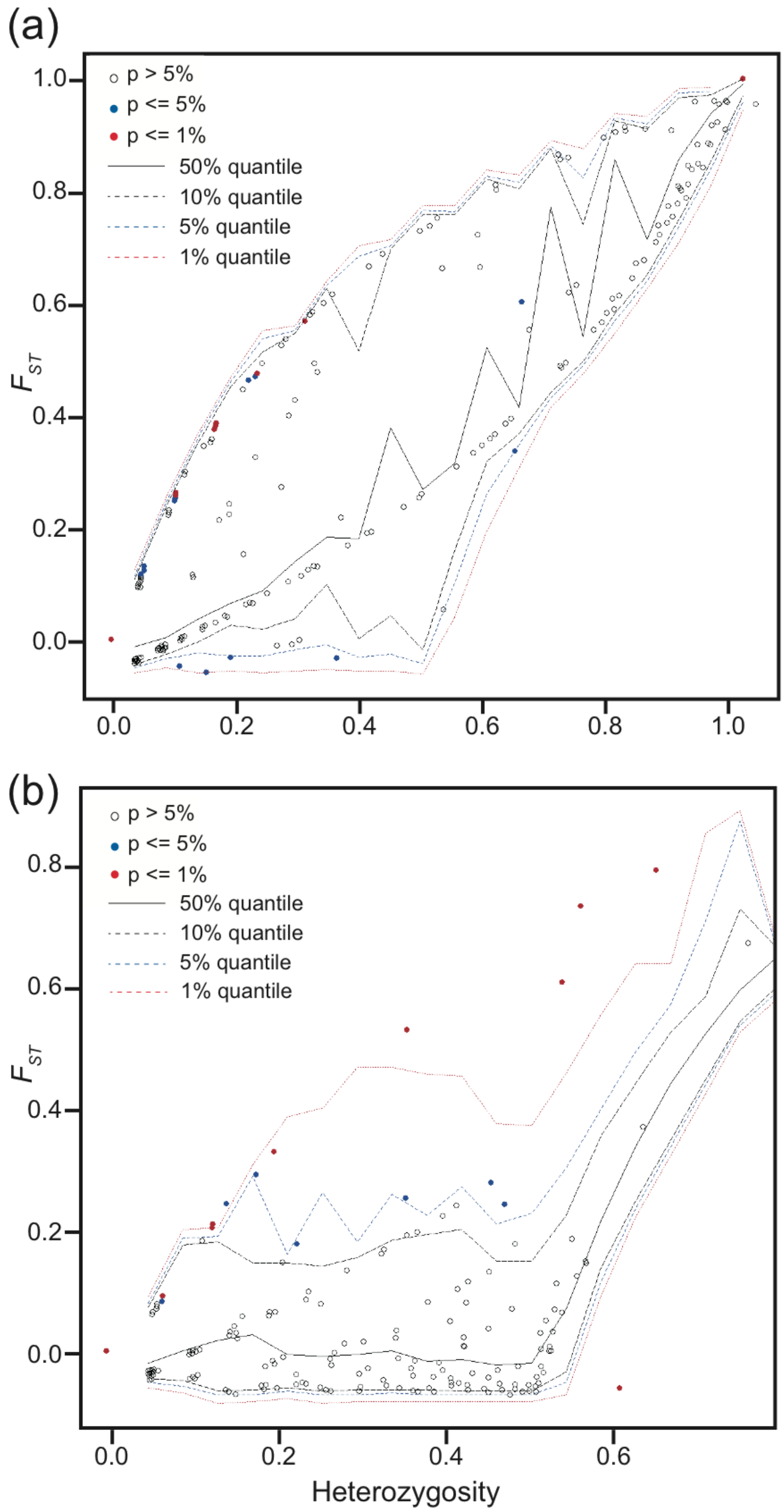

Fig. S4 Results of F-DIST analysis on 1,637 SNP loci. (a) Comparing DT, PY to SH; (b) comparing CH, eastern TH to SH.

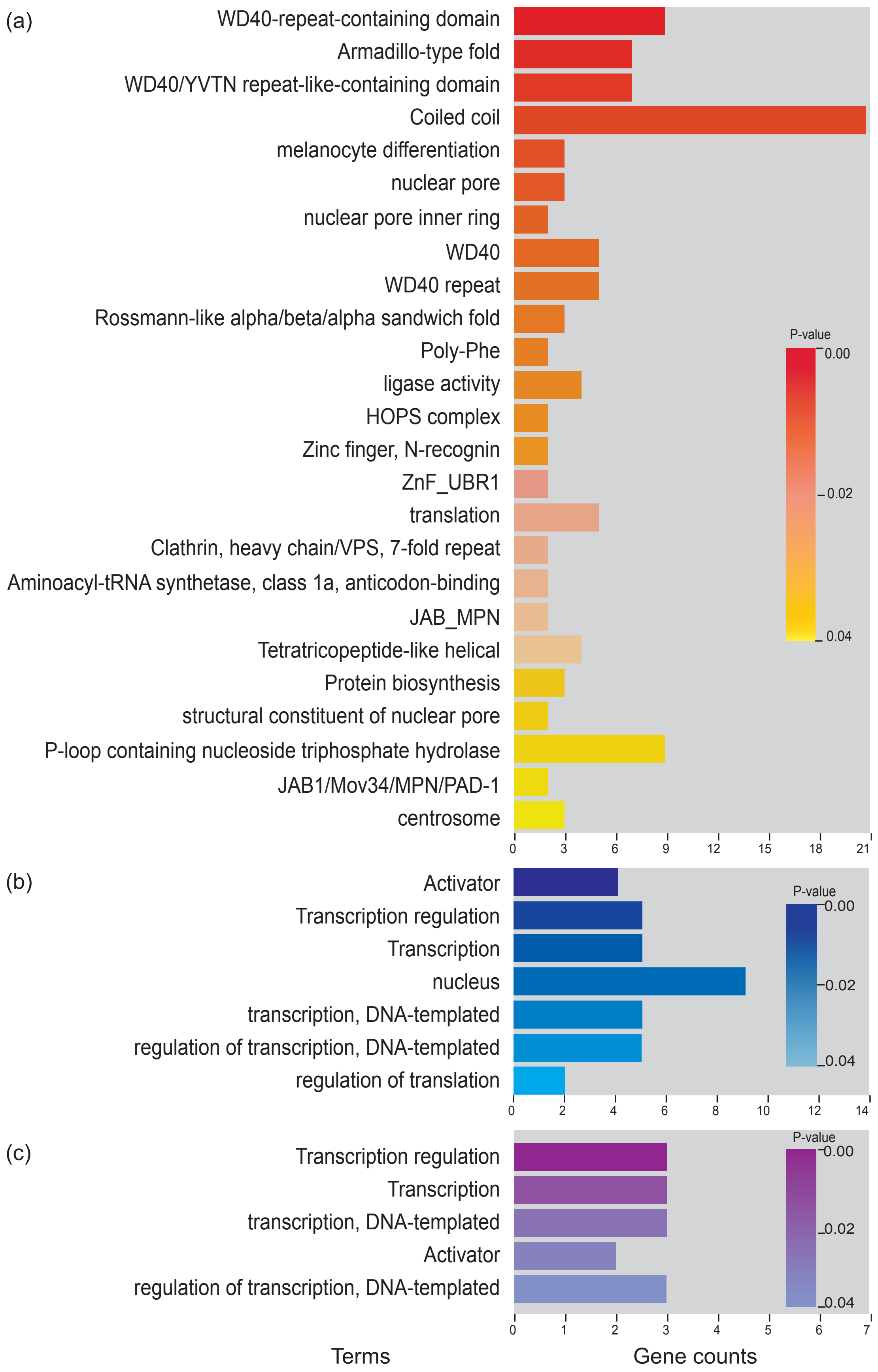

Fig. S5 Functional categorization including biological process, molecular function and cellular component based on gene ontology (GO) annotations of online DIVID. (a) Categories based on 120 disruptive genes comparing DT, PY and SH; (b) categories based on 21 disruptive genes comparing CH, eastern TH and SH; (c) categories based on 9 common genes found in both comparison.
